# The Functional Characterization of TcMyoF Implicates a Family of Cytostome-Cytopharynx Targeted Myosins as Integral to the Endocytic Machinery of Trypanosoma cruzi

**DOI:** 10.1128/mSphere.00313-20

**Published:** 2020-06-17

**Authors:** Nathan Michael Chasen, Menna Grace Etheridge, Ronald Drew Etheridge

**Affiliations:** a Department of Cellular Biology, Center for Tropical and Emerging Global Diseases (CTEGD), University of Georgia, Athens, Georgia, USA; University at Buffalo

**Keywords:** myosin, endocytosis, SPC, cytostome, cytopharynx, reservosome, *Trypanosoma cruzi*

## Abstract

Of the pathogenic trypanosomatids, Trypanosoma cruzi alone retains an ancient feeding apparatus known as the cytostome-cytopharynx complex (SPC) that it uses as its primary mode of endocytosis in a manner akin to its free-living kinetoplastid relatives who capture and eat bacterial prey via this endocytic organelle. In a recent report, we began the process of dissecting how this organelle functions by identifying the first SPC-specific proteins in T. cruzi. Here, we continued these studies and report on the identification of the first enzymatic component of the SPC, a previously identified orphan myosin motor (MyoF) specifically targeted to the SPC. We overexpressed MyoF as a dominant-negative mutant, resulting in parasites that, although viable, were completely deficient in measurable endocytosis *in vitro*. To our surprise, however, a full deletion of MyoF demonstrated only a decrease in the overall rate of endocytosis, potentially indicative of redundant myosin motors at work. Thereupon, we identified three additional orphan myosin motors, two of which (MyoB and MyoE) were targeted to the preoral ridge region adjacent to the cytostome entrance and another (MyoC) which was targeted to the cytopharynx tubular structure similar to that of MyoF. Additionally, we show that the C-terminal tails of each myosin are sufficient for targeting a fluorescent reporter to SPC subregions. This work highlights a potential mechanism used by the SPC to drive the inward flow of material for digestion and unveils a new level of overlapping complexity in this system with four distinct myosin isoforms targeted to this feeding structure.

**IMPORTANCE** The parasite Trypanosoma cruzi is the etiological agent of Chagas disease and chronically infects upwards of 7 million people in the Americas. Current diagnostics and treatments remain grossly inadequate due, in part, to our general lack of understanding of this parasite’s basic biology. One aspect that has resisted detailed scrutiny is the mechanism employed by this parasite to extract nutrient resources from the radically different environments that it encounters as it transitions between its invertebrate and mammalian hosts. These parasites engulf food via a tubular invagination of its membrane, a strategy used by many protozoan species, but how this structure is formed or functions mechanistically remains a complete mystery. The significance of our research is in the identification of the mechanistic underpinnings of this feeding organelle that may bring to light new potential therapeutic targets to impede parasite feeding and thus halt the spread of this deadly human pathogen.

## Abstract (precis)

The parasite Trypanosoma cruzi is the etiological agent of Chagas disease and chronically infects upwards of 7 million people in the Americas. Current diagnostics and treatments remain grossly inadequate due, in part, to our general lack of understanding of this parasite’s basic biology. One aspect that has resisted detailed scrutiny is the mechanism employed by this parasite to extract nutrient resources from the radically different environments that it encounters as it transitions between its invertebrate and mammalian hosts. These parasites engulf food via a tubular invagination of its membrane, a strategy used by many protozoan species, but how this structure is formed or functions mechanistically remains a complete mystery. The significance of our research is in the identification of the mechanistic underpinnings of this feeding organelle that may bring to light new potential therapeutic targets to impede parasite feeding and thus halt the spread of this deadly human pathogen.

## INTRODUCTION

The unicellular protozoan Trypanosoma cruzi is considered to be one of the most significant disease-causing parasites in the Americas. An estimated 7 million people are infected by this parasite, resulting in approximately 50,000 deaths annually (1, 2). Chronic infection by T. cruzi often results in a malady better known as Chagas disease, characterized by the destruction of both smooth and cardiac muscle, causing significant morbidity and mortality in those affected. Currently, there is no effective vaccine against infection and available therapies often fail to cure (3–7). A better understanding of the basic biology of this parasite is essential to being able to identify unique cellular pathways and processes that might be targets for future drug design. Ensuring successful transmission between its vertebrate and invertebrate hosts relies on the ability of T. cruzi to effectively consume host resources in order to grow and propagate; thus, its catabolic capacity and virulence remain inextricably linked (8). Unfortunately, very little is known about how T. cruzi extracts sustenance from the highly disparate environments of its insect and mammalian hosts, but it stands to reason that if its feeding could be inhibited, it would compromise the parasite’s ability to disseminate and cause disease.

Understanding nutrient uptake in T. cruzi has been surprisingly difficult as it does not share the flagellar-pocket-centered endocytic pathway of its more heavily studied pathogenic relatives (T. brucei or *Leishmania* spp.) (9–11). Notably, T. cruzi employs a more ancient mode of endocytosis analogous to what is observed in its monoxenous trypanosomatid relatives (e.g., Paratrypanosoma confusum) or even in free-living bacterivorous kinetoplastids (bodonids) (12). These organisms, like many ciliates, feed via a tubular invagination (cytopharynx) that originates at a cytoplasmic membrane opening (cytostome) and, at least for free-living protozoans, is used to filter feed and engulf prey (13, 14). In T. cruzi, endocytosed material is initially shuttled to late endosome-like storage structures known as reservosomes that, when needed, are converted into digestive lysosomes for the liberation of simple nutrients. This feeding structure, referred to here as the cytostome-cytopharynx complex (SPC) has, strikingly, been completely lost in the closely related dixenous trypanosomatids that infect humans (T. brucei and *Leishmania* spp.) (15). An obvious question emerges from this observation: why was the SPC lost in the salivarian trypanosomatids but retained in T. cruzi? Although speculative, it potentially resulted from the unique environments specifically inhabited by this pathogen: the insect vector hindgut and the mammalian host cytosol (16–18). Because we know so little about how this organelle functions in any protozoan, much less in T. cruzi, we have relied almost entirely on electron microscopy (EM) tomography and three-dimensional (3D) reconstruction work for insights into its basic structure and dynamic behavior. Those studies revealed that the SPC disassembles during division as well as in the infectious stages of the parasite (19, 20). Structurally, those studies were also the first to show that the T. cruzi cytostome-cytopharynx complex utilizes a quartet of microtubule root fibers (which we term “CyQ”) to guide the internal localization of its structure, potentially acting as guiding rails for the transfer and delivery of endocytosed material to the posterior reservosomes (21). The CyQ bundle itself emerges from the basal body region and winds around the flagellar pocket, before interacting with the parasite plasma membrane to form a unique surface prominence known as the preoral ridge (POR) and bridging the flagellar pocket to the cytostome entrance. This bridging POR membrane is both cholesterol and glycan rich and is also nearly devoid of transmembrane proteins. The membrane surface of the POR also displays an intriguing capacity to nonspecifically adhere to a broad range of protein cargoes prior to its being endocytosed via the SPC (22–24). This region of the membrane thus has all the hallmarks of a lipid raft, and it is tempting to suppose that the parasite uses glycosylphosphatidylinositol (GPI)-anchored proteins restricted to this region as receptors to both capture cargo and induce endocytosis via a mechanism analogous to the GPI-linked transferrin receptor of T. brucei (25). At this point in time, however, no cargo receptor proteins have been identified in T. cruzi. In aggregate, these observations suggest that membrane being drawn into the SPC originates at the flagellar pocket and travels up and over the POR, where it binds cargo, and then descends again as it is endocytosed into the internal portion of the SPC (22, 23).

Our lab has previously reported on the first known proteins of this endocytic structure in T. cruzi (26); since that finding, we have begun to systematically analyze the protein composition of the SPC in order to determine the molecular basis for its construction and function. As part of our ongoing search for SPC components, we initiated a bioinformatics-based approach to identify potential novel machinery. With the SPC-targeted proteins from our first published study serving as guides in our bioinformatics-based search, we have been able to quickly identify and assess the targeting of several dozen proteins. From this initial screen, we identified six previously uncharacterized SPC-targeted proteins. Of the six, four were classified as “hypothetical” and localized to the POR, cytopharynx, or microtubule root fibers of the SPC (named here CP4 through CP7). The remaining two were cytopharynx restricted and were previously reported as orphan myosins (MyoF and MyHd) in an in-depth phylogenetic analysis of the myosin gene family in trypanosomatids (27). Because MyHd was predicted to be a nonfunctional myosin, we focused on assessing the function of MyoF as it would have the potential to explain how this organelle actively brings in material for digestion. Using a catalytically dead MyoF dominant-negative overexpression line, we observed that parasites were completely devoid of measurable endocytosis. A direct knockout (KO) of MyoF, however, merely caused a reduction in the endocytic rate, indicating that other myosin motors either may be compensating for the loss of MyoF or are operating redundantly within the SPC endocytic system. With this in mind, we localized all remaining orphan myosin motors of T. cruzi and identified three additional myosins (MyoB/C/E) that targeted to distinct subregions of the SPC (27). We also showed that the C-terminal tails of each motor are sufficient for targeting a fluorescent reporter to the SPC structure. Overall, this work increases our understanding of the SPC organelle’s mechanistic underpinnings and suggests an interesting dichotomy in the organization of the cytostome-cytopharynx complex, where sets of molecular motors are targeted to either the POR or the cytopharynx, with each potentially serving a specialized role in the proper functioning of this endocytic organelle.

## RESULTS

### Identification of novel cytostomal machinery.

**FIG 1:**
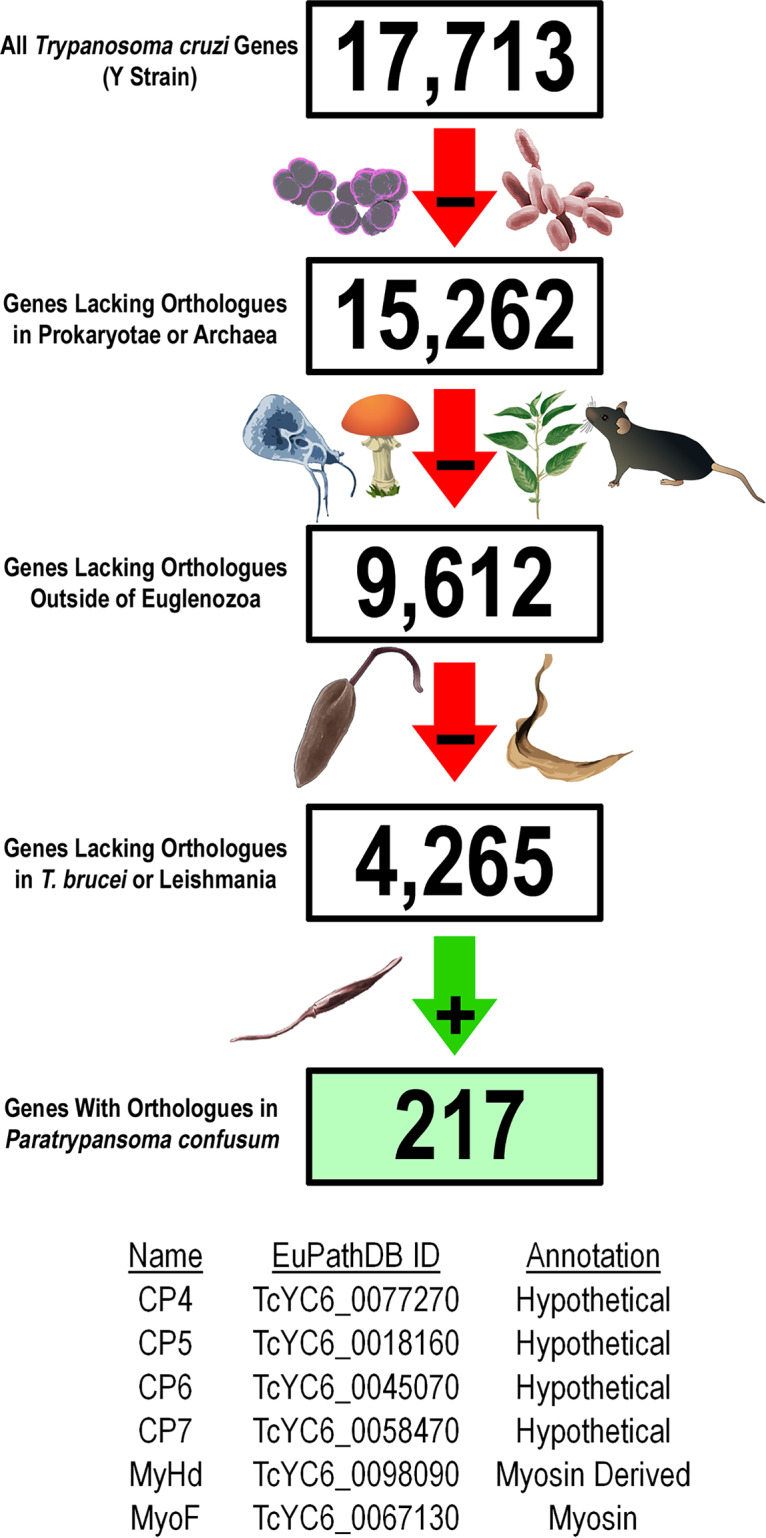
(Top panel) Bioinformatic identification of potential SPC proteins. The list of 17,713 annotated T. cruzi genes from the Y strain genome assembly was narrowed down to 9,612 genes that do not have orthologues outside the Euglena genus. This list was further trimmed to 4,265 genes by eliminating all genes that have orthologues in *Leishmania* and T. brucei which lack the SPC. The final filter eliminated genes that did not have orthologues in the monoxenous kinetoplastid parasite Paratrypanosoma confusum, which has an SPC, resulting in a list of 217 potential SPC genes. (Bottom panel) List of seven proteins that have been shown to localize to the SPC. ID, identifier.

**FIG 2:**
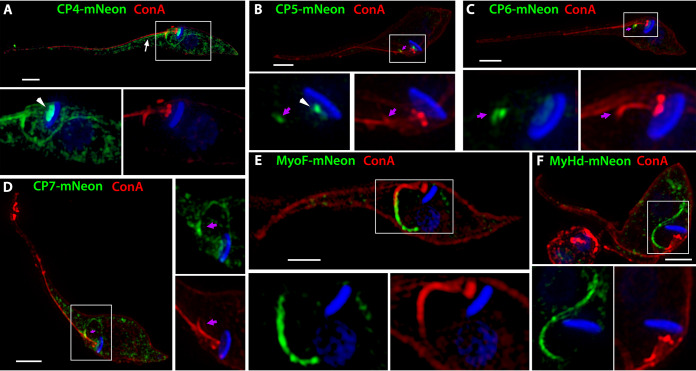
Identification and localization of six SPC proteins. (A) CP4 localizes to the SPC, potentially on the CyQ and MtQ (arrow) microtubule root fibers and the basal bodies associated with the flagellum (arrowhead). (B to D) CP5 (B), CP6 (C), and CP7 (D) localize to the POR of the SPC labeled by the lectin concanavalin A (purple arrows). CP5 also shows basal body localization (arrowhead). (E and F) Two proteins annotated to contain myosin motor domains (MyoF [E] and MyHd [F]) localize to the cytopharynx of the SPC. Nuclei and kinetoplasts in all fluorescent images were stained with DAPI (4′,6-diamidino-2-phenylindole) (blue). Scale bars, 2 μM.

**FIG 3:**
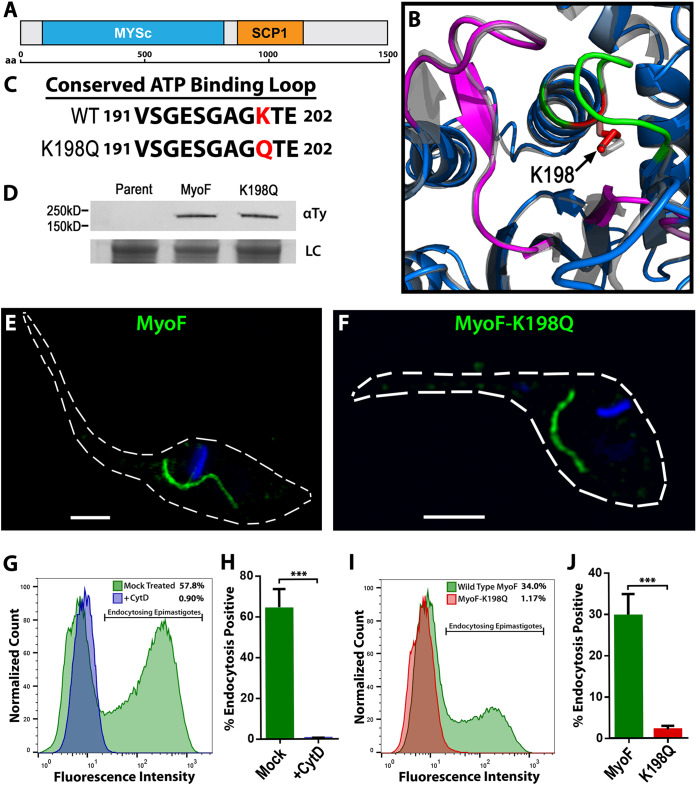
Dominant-negative MyoF mutants are defective in SPC endocytosis. (A) Schematic of the *MyoF* gene showing the annotated myosin and SCP1 coiled-coil domains. (B) I-TASSER structure prediction of MyoF (colors) aligned with a known myosin crystal structure (gray) showing ATP binding P-loop region (green) and switch regions (magenta). A conserved lysine residue that is essential for P-loop function is shown in red (K198, arrow). (C) Sequence of the ATP binding P-loop region showing the K198 amino acid residue (red) that was mutated to a glutamine (K198Q) to generate a rigor mutant of MyoF (MyoF-K198Q) for dominant-negative expression. (D) Immunoblot probed with anti-Ty antibody showing similar levels of MyoF and MyoF-K198Q in the chosen subclones. LC, loading control. (E and F) Overexpressed MyoF (E) and MyoF-K198Q (F) fused to mNeon exhibited the same localization in epimastigotes. (G and H) Flow cytometry of epimastigotes after fluorescent BSA feeding shows that the actin inhibitor cytochalasin D prevents SPC endocytosis. (I and J) Flow cytometry of epimastigotes after fluorescent BSA feeding shows a dominant-negative endocytosis defect in the MyoF-K198Q mutants. G and I, counts; H and J, percent positive. Nuclei and kinetoplasts in all fluorescent images were stained with DAPI (blue). Scale bars, 2 μM.

As part of our ongoing efforts to characterize the enigmatic SPC feeding apparatus, we aimed to identify novel SPC proteins using a bioinformatics-based strategy, taking advantage of the evolutionary loss of this organelle in the salivarian trypanosomatid lineages. In order to generate a manageable T. cruzi candidate gene list, we first removed potential orthologues from prokaryotes, archaea, and any eukaryote outside Euglenozoa. The resulting list was further reduced by eliminating T. cruzi genes with orthologues in the SPC-less *Leishmania* spp. and T. brucei. Finally, we focused only on those genes also present in the SPC-containing monoxenous trypanosomatid Paratrypanosoma confusum, resulting in a list of 217 genes (Fig. 1). Our localization screens from this list identified four hypothetical proteins (CP4 to CP7) and two intriguing proteins with predicted myosin domains (MyHd and MyoF) that we localized to the SPC in both epimastigotes (Fig. 2) and amastigotes (see Fig. S1A to E in the supplemental material) using a C-terminal mNeon fusion and the pTREX overexpression vector (28). With the CP4, CP5, CP6, and MyoF-mNeon overexpression lines, we also demonstrated colocalization with our SPC foundational marker, CP1 (Fig. S1F, G, H, and I, respectively). It is worth noting that these identified myosin motors are the first enzymatic components potentially associated with the SPC and had previously been designated as orphan myosins, as they were selectively lost in the SPC-less parasitic trypanosomatids (27). The CP4 protein (TcYC6_0077270) localized to structures reminiscent of the microtubule root fibers associated with the flagellar attachment zone (arrow) and cytostomal microtubule quartet rootlets (Fig. 2A; see also Fig. S1A and B) (15). Staining of CP4-mNeon-expressing parasites with antibodies (Abs) for the T. cruzi flagellar marker FCaBP highlighted the parallel orientation of CP4 relative to the parasite flagellum (Fig. S2A) (29). A portion of CP4 also colocalized with the basal body marker RP2 using antibodies originally generated for the closely related T. brucei homologue (Fig. S2B) (30). This suggested that CP4 may have a more generalized affinity for microtubule structures in the parasite. In our previous work (26), we demonstrated that we can highlight the cytostome entrance known as the preoral ridge (POR) region of the parasite using the fluorescently conjugated lectin concanavalin A (ConA) and fluorescence microscopy. With this marker, we observed that CP5 (TcYC6_0018160), CP6 (TcYC6_0045070), and CP7 (TcYC6_0058470) localized to the POR (Fig. 2B to D; see also Fig. S1C and D). As with CP4, a portion of the signal for CP5, which also contains a predicted Kelch motif (IPR015915), and for CP7 colocalized with the basal body marker as well (Fig. S2D and C, respectively) (31). The MyoF and MyHd isoforms both contained predicted myosin motor head domains (MYSc_2a) and were shown to localize to the internal tubular (cytopharynx) portion of the SPC (Fig. 2E and F; see also Fig. S1E). Unlike MyoF, however, MyHd likely does not possess an active myosin domain as it lacks the conserved ATP binding P-loop region (32), resulting in this gene’s designation as a myosin H-derived protein (27). Although potentially an artifact due to overexpression, we noted that in SPC-less dividing epimastigotes, MyHd-mNeon showed filamentous labeling of an unusually enlarged posterior structure (Fig. S2E). MyoF does contain an intact myosin domain along with a synaptonemal complex 1-like (SCP1) coiled-coil domain (Fig. 3A); thus, we focused on MyoF as a protein with the potential to be directly involved in the mechanical activity of endocytosis in T. cruzi.

10.1128/mSphere.00313-20.1 FIG S1 Identified proteins label the SPC in amastigotes. (A) CP4 shows localization in amastigotes similar to that seen in epimastigotes, with basal body and potential microtubule root fiber labeling. (B) The localization of CP4 in a dividing amastigote labeling both basal body regions (asterisks) and, potentially, the associated microtubule root fibers (arrow). (C and D) CP5 and CP6 both show localization to the preoral ridge (purple arrow) in front of the cytopharynx labeled by anti-CP1 (1:1,000). CP5 also shows the basal body localization observed in epimastigotes (arrowhead). CP6 often shows additional posterior labeling (arrowhead) that, due to its unusual brightness, may represent an overexpression artifact. (E) MyoF shows SPC cytopharynx labeling consistent with the epimastigote localization. (F, G, H, and I) IFAs using anti-CP1 show the SPC localizations of CP4-mNeon (F), CP5-mNeon (G), CP6-mNeon (H), and MyoF-mNeon (I). Nuclei and kinetoplasts in all fluorescent images were stained with DAPI (blue). Scale bars, 2 μM. Download FIG S1, PDF file, 0.6 MB. Copyright © 2020 Chasen et al. 2020 Chasen et al. This content is distributed under the terms of the Creative Commons Attribution 4.0 International license.

10.1128/mSphere.00313-20.2 FIG S2 Colocalization of SPC proteins with other cellular markers. (A) CP4-mNeon shows labeling adjacent to the flagellar marker anti-FCaBP. (B, C, and D) CP4-mNeon (B), CP5-mNeon (D), and CP7-mNeon (C) all show some labeling of the basal body region labeled by anti-TbRP2. (E) In dividing epimastigotes, MyHd-mNeon shows a filamentous labeling of an unusually elongated posterior structure. These MyHd-mNeon-expressing epimastigotes did not survive drug selection. Nuclei and kinetoplasts were stained with DAPI (blue). Scale bars, 2 μM. Download FIG S2, PDF file, 0.1 MB. Copyright © 2020 Chasen et al. 2020 Chasen et al. This content is distributed under the terms of the Creative Commons Attribution 4.0 International license.

### Dominant-negative MyoF mutants have a severe defect in SPC endocytosis.

To assess the role of MyoF in the endocytic function of the SPC, we first pursued a dominant-negative strategy to elucidate its contribution to this process. To generate dominant-negative mutants, we overexpressed a version of MyoF with a lysine-to-glutamine substitution in the ATP-binding P-loop at position 198 (K198Q, Fig. 3B and C). Previous research on myosins had demonstrated that this mutation results in motors that suffer from a “rigor” phenotype and thus are unable to dissociate from actin filaments. These “dead” myosins therefore could compete for actin binding sites with endogenous MyoF and thus block motor progression (33). Overexpressed MyoF-K198Q was shown to target properly to the cytopharynx structure and was expressed at levels similar to the wild-type overexpression (Fig. 3D to F). To assess endocytic function, we incubated parasites with fluorescently labeled bovine serum albumin (BSA) followed by an analysis of the rate of uptake after 30 min of feeding. Wild-type parasites growing in log phase consistently showed two defined peaks, with the first peak representing dividing parasites which break down the SPC and the second being the actively feeding population. Additionally, we utilized the actin inhibitor cytochalasin D (CytD) as a negative control for complete ablation of endocytosis as has been shown previously with the functionally similar cytochalasin B (34, 35) (Fig. 3G and H). Comparing parasites overexpressing wild-type MyoF-mNeon to the dominant-negative mutants, we observed a near complete loss of endocytic function in these parasites (Fig. 3I and J). Although the effects were milder, it should also be noted that overexpression of the wild-type MyoF-mNeon and even a motorless C-terminal portion of MyoF-mNeon (Fig. S3A) also reduced the endocytic rate of parasites, suggesting that overexpression of the SPC components could itself impact endocytosis albeit not to the same extent as that seen with the MyoF-K198Q mutant. Overall, this implied that the catalytic activity of MyoF plays a critical role in the ability of T. cruzi to pull in cargo via its cytostome-cytopharynx complex but also revealed an unanticipated conundrum; the parasites were able to survive and grow in culture at rates on par with wild-type parasites despite the absence of measurable endocytic function (Fig. S3B). This phenomenon suggested that we would be able to generate gene deletion knockouts of essential endocytic components and analyze SPC function directly without impacting parasite fitness in culture.

10.1128/mSphere.00313-20.3 FIG S3 Endocytosis assays of C-terminal MyoF overexpression mutants and MyoF dominant-negative growth curve. (A) Representative endocytosis assay shows that overexpression of the C-terminal region of MyoF (aa 904-1481) fused to the C terminus of mNeon (*MyoF-Ct*) was sufficient to cause a dominant-negative effect on endocytosis. (B) Growth assay showing that expression of dominant-negative MyoF-K198Q did not have a deleterious effect on growth in LIT media. Download FIG S3, PDF file, 0.3 MB. Copyright © 2020 Chasen et al. 2020 Chasen et al. This content is distributed under the terms of the Creative Commons Attribution 4.0 International license.

### Deletion of MyoF results in a reduced rate of SPC-mediated endocytosis.

**FIG 4:**
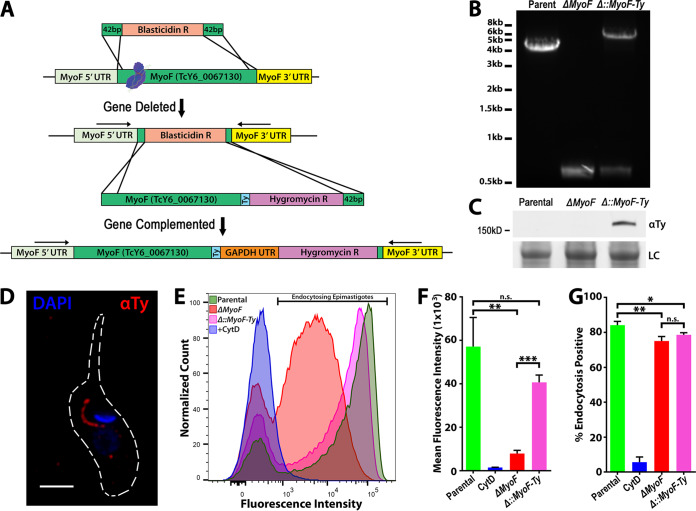
MyoF deletion mutants exhibit a reduction in the rate of SPC endocytosis. (A) Scheme for CRISPR/Cas9 gene replacement and complementation strategy used to generate Δ*MyoF* deletion and complemented (Δ::*MyoF*-*Ty*) mutants. GAPDH, glyceraldehyde-3-phosphate dehydrogenase. (B) PCR amplification of the genomic locus showing replacement of both parental loci (high-molecular-weight [MW] band) with the blasticidin resistance gene (low-MW band) in a subclone of the Δ*MyoF* mutant. PCR of a nonsubcloned population of complemented parasites shows insertion of the MyoF-Ty-Hygro repair template into the *MyoF* locus. (C) Anti-Ty immunoblot of lysates from parental, Δ*MyoF*, and (Δ::*MyoF*-*Ty*) epimastigotes showing expression of MyoF-Ty in the complemented parasites. (D) Immunofluorescence assays of a (Δ::*MyoF*-*Ty*) epimastigote showing expression and proper SPC localization of MyoF-Ty. (E) Flow cytometry analysis of epimastigotes fed fluorescent BSA showing a reduced rate of feeding in Δ*MyoF* mutants, a phenotype that is partially rescued in the complemented (Δ::*MyoF*-*Ty*) mutants. (F) Quantification of the feeding rate results represented by reduced mean fluorescence of endocytosing epimastigotes. A dramatic reduction in the feeding rate was seen in the Δ*MyoF* mutants, which was partially rescued by complementation (Δ::*MyoF-Ty*). (G) A minor but statistically significant percentage of the total number of Δ*MyoF* and (Δ::*MyoF*-*Ty*) epimastigotes showed reduced levels of endocytosed fluorescent BSA during the assay.

As stated previously, endocytosis was undetectable in the dominant-negative MyoF overexpression mutants with no apparent impact on growth or viability; thus, we attempted to generate homozygous deletion mutants of MyoF to enable us to perform a more detailed analysis of its role in parasite feeding. Using a CRISPR/Cas9-mediated gene deletion strategy (Fig. 4A schematic; see also Fig. S4B), we induced double-stranded breaks followed by homology-mediated repair to replace the coding region of MyoF with the blasticidin resistance marker (36). Following drug selection and clonal isolation, homozygous deletion mutants were identified using diagnostic PCR to identify the modified locus using primers that amplify from the 5′ and 3′ untranslated regions (UTR) of the MyoF gene. Using this method, we were able to distinguish parental, heterozygous, and homozygous knockout (KO) clones. Prior work to generate homozygous double deletion mutants in T. cruzi required the use of two different drug markers, one per gene locus; however, using the CRISPR/Cas9 strategy, we can drive a single drug marker into both alleles assuming there is no severe decrease in fitness or viability (Fig. 4B, diagnostic PCR). In order to demonstrate that the altered endocytosis was indeed due to a loss of MyoF, we also complemented this gene back into its endogenous locus using CRISPR/Cas9 targeting of the blasticidin drug marker and its replacement with a Ty-tagged version of the MyoF gene as well as a hygromycin resistance marker (Fig. 4A, schematic). A diagnostic PCR and Western blot analysis of the complemented MyoF-Ty were performed to demonstrate the presence of an altered genetic locus and correct protein size (Fig. 4B and C), while immunofluorescence assays (IFAs) showed proper localization to the SPC as well (Fig. 4D). The resulting MyoF knockout strain (Δ*MyoF*) was then compared to the parental strain, complement (Δ::*MyoF-Ty*) strain, and CytD control strain in our flow cytometry-based endocytosis assay. We observed an 86% reduction in the rate of endocytosis in the Δ*MyoF* mutant (Fig. 4E; quantified in panel F), which was a much less severe phenotype than the nearly complete ablation of BSA-647 uptake seen in the dominant-negative overexpression line (Fig. 3I and J). This endocytic rate defect was also shown to be restored in the complemented line (Fig. 4E; quantified in panel F). A small but significant change in the percentage of cells actively endocytosing was also observed (Fig. 4G). This suggested that the catalytically dead MyoF-K198Q protein was poisoning other aspects of endocytosis separate from the direct function of MyoF and that SPC-mediated uptake was likely driven by redundant mechanisms such as additional myosin motors. We also generated deletion mutants of CP1 and CP2 (26) (Fig. S5A and B) which exhibited 35% and 40% reduced feeding rates, respectively (Fig. S5C; quantified in panel D). A minor reduction in the total number of parasites undergoing endocytosis was also seen in the Δ*MyoF* and Δ*cp2* mutants but not the Δ*cp1* mutants (Fig. S5E). During replication, the SPC is disassembled and endocytosis does not occur (19), suggesting the possibility that Δ*MyoF* and Δ*cp2* mutants may spend increased time in those stages of the cell cycle.

10.1128/mSphere.00313-20.4 FIG S4 Plasmid constructs generated and used in this work. (A) Schematic of the pMiniTREX vector based on the picoZ minimalist vector, combined with elements of pTREX and a new prostaglandin F2alpha synthase (TcYC6_0070460) intergenic region used for the selection marker (instead of the usual repeat of the GAPDH [glyceraldehyde-3-phosphate dehydrogenase] intergenic region). (B) Schematic of the pMiniTREX-spCas9 vector lacking a selection cassette for transient expression of spCas9. (C) Schematic of the pTMiniTREX, which contains the Bba_B1006 terminator upstream of the insertion site, to reduce any potentially toxic expression of cloned products during cloning from cryptic bacterial promoters in the HX1 intergenic region. (D) Schematic of the pTMiniTREX-spCas9 vector with selection cassette. Download FIG S4, PDF file, 0.2 MB. Copyright © 2020 Chasen et al. 2020 Chasen et al. This content is distributed under the terms of the Creative Commons Attribution 4.0 International license.

10.1128/mSphere.00313-20.5 FIG S5 Δ*cp1* and Δ*cp2* deletion verification and representative endocytosis assay. (A and B) PCR amplification of the genomic locus of CP1 (A) and CP2 (B) showing replacement of both parental loci (high-MW band) with the blasticidin resistance gene (low-MW band). (C) Representative endocytosis assay of Δ*cp1* and Δ*cp2* mutants. (D) Quantification of the feeding rate results represented by reduced mean fluorescence of endocytosing epimastigotes. A dramatic reduction in the feeding rate was seen in the Δ*MyoF* mutants, and a reduction in the feeding rate was also observed in the Δ*cp1* and Δ*cp2* epimastigotes. (E) A significantly reduced percentage of both Δ*MyoF* and Δ*cp2* epimastigotes endocytosed BSA during the feeding assays. This defect was not observed in Δ*cp1* epimastigotes. Download FIG S5, PDF file, 0.7 MB. Copyright © 2020 Chasen et al. 2020 Chasen et al. This content is distributed under the terms of the Creative Commons Attribution 4.0 International license.

### Four distinct orphan myosin isoforms target to the SPC endocytic structure.

**FIG 5:**
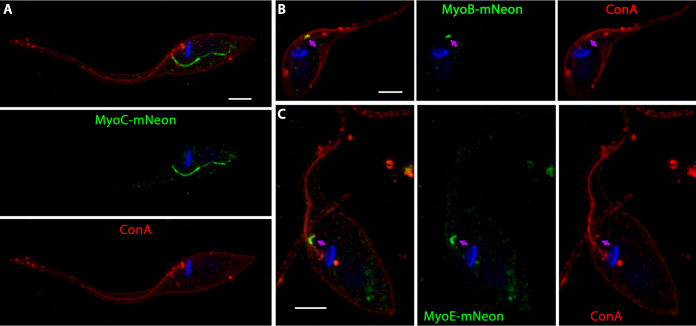
Three additional T. cruzi myosins localize to the SPC. (A) Overexpression and superresolution structured illumination (SR-SIM) microscopy of MyoC-mNeon revealed cytopharynx localization, suggesting that MyoC may provide redundancy to the cytopharynx endocytic machinery. (B and C) MyoB (B) and MyoE (C) both localize to the preoral ridge of the SPC (purple arrows). Nuclei and kinetoplasts in all fluorescent images were stained with DAPI (blue). Scale bars, 2 μM.

To determine if the observed discrepancy in endocytic rates between the Δ*MyoF* knockout and the dominant-negative MyoF mutants was due to functional redundancy by additional unknown myosins, we proceeded to localize all the remaining orphan myosins of T. cruzi originally identified bioinformatically by de Sousa et al. (27). We suspected that a built-in redundancy in this system may exist in a manner analogous to what has been observed in a variety of vesicular trafficking systems (37, 38). Using our C-terminal mNeon fusion screening methodology and a new minimal pTREX vector that we named pMiniTrex (Fig. S4A), we cloned seven additional myosin isoforms: MyoA1 (TcYC6_0002820), MyoA2 (TcYC6_0004170), MyoB (TcYC6_0044730), MyoC (TcYC6_0123790), MyoD (TcYC6_0062870), MyoE (TcYC6_0066350), and MyoG (TcYC6_0075240). Epimastigote parasites were then transfected with fusion constructs followed by staining with ConA and imaging using fluorescence microscopy. From this screen, we determined that, while four of the seven orphan myosins showed a diffuse cytosolic localization (Fig. S6A to D), three of them targeted to the SPC: MyoB, MyoC, and MyoE (Fig. 5). In a pattern similar to what we see with MyoF, MyoC also appeared to target to the cytopharynx tubule (Fig. 5A). Curiously, however, MyoB and MyoE appeared to localize not to the cytopharynx tubule but rather to a subregion of the SPC we refer to as the preoral ridge (POR). This POR targeting hypothesis is supported by the colocalization of MyoB and MyoE with ConA staining (Fig. 5B and C). Interestingly, a dichotomous pattern has thus emerged in the substructure of the SPC, with proteins appearing to target generally to either one of two places: the tubular cytopharynx structure or the preoral ridge. The functional significance of these localization patterns, however, remains to be determined.

10.1128/mSphere.00313-20.6 FIG S6 Cytosolic labeling of the other orphan myosins in epimastigotes. SR-SIM imaging of both MyoA isotypes (A and B), MyoD (C), and MyoG (D) showed only diffuse cytosolic labeling. Nuclei and kinetoplasts in all fluorescent images were stained with DAPI (blue). Scale bars, 2 μM. Download FIG S6, PDF file, 0.2 MB. Copyright © 2020 Chasen et al. 2020 Chasen et al. This content is distributed under the terms of the Creative Commons Attribution 4.0 International license.

### The tail domain of each SPC myosin isoform is sufficient to target mNeon to the SPC.

**FIG 6:**
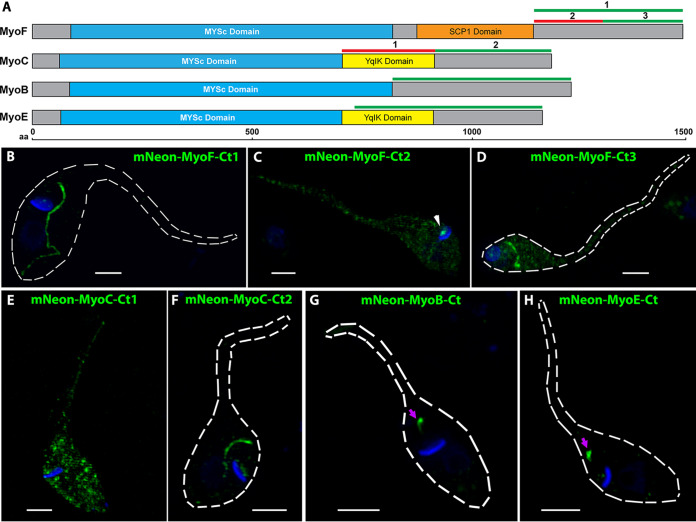
The C-terminal tail regions of myosin F, C, B, and E are sufficient for SPC targeting. (A) Schematic of myosin genes and the regions (green and red lines) that were fused to the C terminus of mNeon for localization. The green-colored regions were sufficient for localization to the SPC when fused to mNeon, whereas the red-colored regions were not. (B, C, and D) The MyoF-Ct1 (B) and MyoF-Ct3 (D) regions were sufficient to target mNeon to the SPC cytopharynx, although MyoF-Ct3 had a notably less defined localization. MyoF-Ct2 (C) was not sufficient for SPC targeting but did demonstrate localization adjacent to the kinetoplast (arrowhead). (E and F) MyoC-Ct2 (F) was sufficient for targeting mNeon to the SPC cytopharynx, whereas MyoC-Ct1 (E) was not. (G and H) The C-terminal regions of MyoB (G) and MyoE (H) were both sufficient to target mNeon to the SPC preoral ridge. Nuclei and kinetoplasts in all fluorescent images were stained with DAPI (blue). Scale bars, 2 μM.

Our observations indicating that two of the SPC myosin isoforms targeted to the tubular invagination whereas two others trafficked to the preoral ridge region were noteworthy and implied a high degree of restrictive organization in these sectors of the endocytic structure. As is seen in other model organisms, there are often multiple myosin isoforms that operate in semisegregated locations within an individual cell, and although many of these molecular motors contain the standard motor head, linker/hinge, and dimerization coil domains, the most divergent areas of the protein are often located at the C termini. These regions, referred to as the cargo binding domains, are largely responsible for the subcellular localization of different isoforms (39–41). This, in turn, gives the cell the ability to specialize enzymatic machinery with similar capacities, e.g., that of walking on actin filaments, and to target them to engage with distinct molecular components in various areas of the cells. To assess the location of the SPC targeting information, we cloned the C-terminal region, after the motor head domain of each myosin isoform (summarized in Fig. 6A), and fused it to a constitutively expressed mNeon reporter in our modified bacterial terminator-containing pTMiniTrex vector to prevent potentially toxic expression during cloning from cryptic bacterial promoters in the pTREX HX1 intergenic region (Fig. S4C). To investigate the MyoF C-terminal tail, we first cloned the sequence downstream of the SCP1 domain (amino acids [aa] 1145 to 1481). Using this sequence (MyoF-Ct1), mNeon targeted to the SPC in a manner similar to that seen with full-length MyoF (Fig. 6B), demonstrating that this region is sufficient for targeting to the SPC cytopharynx. To further narrow down the targeting region, we split this portion into two halves, MyoF-Ct2 (aa 1145 to 1302) and MyoF-Ct3 (aa 1303 to 1482), to test their ability to target mNeon to the SPC. While the N-terminal half, MyoF-Ct2, showed mislocalization to a kinetoplast DNA (kDNA)-adjacent region (Fig. 6C, arrowhead), the MyoF C-terminal half, Myo-Ct3, was sufficient for cytopharynx targeting of mNeon (Fig. 6D), albeit with a signal that was more diffuse than that observed with MyoF-Ct1, suggesting that both parts of the C-terminal domain likely contribute to efficient localization. We approached MyoC in a similar manner, demonstrating that the C-terminal tail region, but not the Yqik domain, was sufficient for targeting mNeon to the SPC cytopharynx (Fig. 6E and F). Likewise, the C-terminal regions of both MyoE and MyoB were able to target mNeon to the SPC preoral ridge (Fig. 6G and H). In total, these observations suggest that the restricted localization that we see for each myosin is most likely due to its C-terminal interaction with the distinct SPC machinery preferentially localized to these regions. The identity of these interaction partners and the minimal domain or motif required for targeting will be the subject of future investigations.

## DISCUSSION

In our previously published study on the T. cruzi cytostome-cytopharynx complex (SPC) (26), we identified the first known protein components of the SPC in this or any organism and demonstrated that the SPC protein CP1 follows a pattern of altered expression and localization when this structure breaks down during division and infectious-stage transformation. This confirmed what had been observed structurally in prior EM tomography studies, and we assume that this is most likely a general pattern for most SPC proteins (19, 20). We initially, and without success, sought to identify, through protein interaction studies, specific molecular machinery that could provide some functional insight into how this organelle draws in sustenance from the extracellular milieu. As an alternative, we decided to leverage our identification of the SPC components, CP1, CP2, and CP3, to perform a bioinformatics-based search. Using the genomic sequences of distantly related kinetoplastids, as well as T. cruzi transcriptional data archived in the kinetoplastid TriTryp database (tritrypdb.org), we built filtering criteria to predict potential SPC components. From the resulting narrowed list of over 200 genes, we prioritized those that were highly expressed during the replicative stages of the parasite when the SPC is active and focused on enzymatic proteins as they had the potential to shed light on how this structure operates mechanistically. In this list, we observed the presence of two myosin domain-containing proteins (MyoF and MyHd) that were of particular interest since it is well known that enzymatic motor proteins play critical roles in vesicular trafficking in eukaryotes and also because it had been observed that endocytosis in T. cruzi is severely affected by cytochalasins, inhibitors of the actin filament substrate of myosins (34, 35). These myosins also caught our attention because they are present in the SPC-containing T. cruzi but were lost in the SPC-less pathogenic trypanosomatids: T. brucei and *Leishmania* spp. Thus, we speculated that these enzymes may have been discarded when the SPC was abandoned as a primary mode of endocytosis (27).

We initially subjected the two orphan myosins and various hypothetical proteins to localization screening and demonstrated that the active myosin (MyoF) and the pseudomyosin (MyHd) and four additional hypothetical proteins targeted to subregions of the SPC. Our continued work on this structure has highlighted three major regions of the SPC where proteins regularly target: the cytopharynx (tubular invagination), the preoral ridge (the region between the cytostome entrance and flagellar pocket), and the less-well-defined microtubule root fibers (guiding the cytoskeletal structure upon which the SPC is built) (21). CP4, we believe, localizes to the latter, although well-characterized markers for root fibers in T. cruzi are lacking. CP5, CP6, and CP7 have an evident focal localization at a region that coincides with the POR (22, 23, 26, 42, 43). MyoF and MyHd, on the other hand, presented in much the same way as CP1 did, as long linear structures that descend deep into the parasite body, a characteristic of the cytopharynx (26). How the cytostome-cytopharynx complex actually pulls in cargo-laden vesicles to be digested was not known; thus, we decided to focus on the predicted functional myosin (MyoF) to determine if this motor played an active role in the endocytic process. By overexpressing a catalytically dead rigor mutant of MyoF (MyoF-K198Q), we were given the first hint that myosins play an important role in normal SPC function (33, 44). These mutants, while viable and with normal growth kinetics, demonstrated nearly undetectable levels of endocytosis that mirrored what we saw with CytD-treated controls, a counterintuitive observation that conflicted with our original hypothesis that SPC-mediated endocytosis was essential for parasite growth.

It quickly became clear that a lack of endocytosis had no adverse effect on axenic growth of epimastigote parasites in culture; therefore, we were forced to reconsider the role that this organelle plays in nutrient acquisition and to ask an obvious question: what then is the purpose of cytostome-cytopharynx complex in the parasite life cycle? Although preliminary and outside the scope of this study, we have observed that epimastigote mutants deficient in endocytosis demonstrate an acute sensitivity to starvation conditions that are easily survived by wild-type parasites. We find this interesting because starvation represents, in many ways, an environmental eventuality anticipated by the parasite in the insect hindgut and also serves as a robust trigger for metacyclic-stage transition (i.e., metacyclogenesis) (16, 45, 46). As a result, we hypothesize that the SPC may, in fact, be part of the transmission strategy used by epimastigotes to persist in the insect vector hindgut during the long periods, often a month or more, between blood meals. It is possible that cytostome-based feeding could serve as T. cruzi’s solution to long-term macromolecular nutrient storage. This is an appealing hypothesis since other modes of classical carbohydrate storage, such as glycogen in animal cells or amylopectin in apicomplexans, have not been observed in this parasite and thus it remains unknown how T. cruzi survives long periods of nutrient scarcity (47–49). T. cruzi, it seems, may rely on SPC-mediated endocytosis in order to stockpile engulfed proteins and lipids inside the dense vesicular depot known as the reservosome (50–53). Work by others has shown that when nutrients become limited, T. cruzi activates its autophagy pathway, resulting in the acidification and activation of cysteine proteases in its reservosomes and thereupon converting them from late endosome-like vesicles into digestive lysosomes (46, 54). Additionally, metabolomic studies have demonstrated that under starvation conditions, parasites transition their metabolism away from glycolysis and become reliant on amino acid catabolism for energy production. Proteins and lipids digested in reservosomes could, theoretically, provide the raw materials needed to support this marked metabolic shift (55). Thus, it has been seen that by the end of metacyclogenesis, there is a pronounced decrease in the number of reservosome structures as they appear to be consumed to support stage conversion and maintain parasite viability (56). The functional role of the SPC is an important question that we will address in greater detail as we continue to dissect the nature of this endocytic organelle and its role in the various stages of the T. cruzi life cycle.

**FIG 7:**
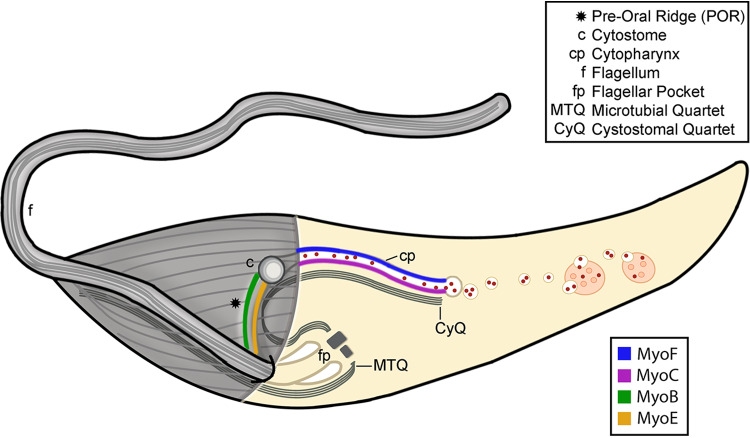
A family of orphan myosins target to distinct regions of the SPC. A cartoon schematic of a T. cruzi epimastigote is shown, highlighting the internal and external structures of the cytostome-cytopharynx complex. The microtubules of the axoneme, cytoskeleton, and root fibers are depicted in gray. The individual components of the SPC and parasite body are referenced in the associated key. Different colored lines denote the observed locations of the four orphan myosins targeted to distinct subregions of the cytostome-cytopharynx complex.

Since endocytosis did not appear to be critical for parasite viability in culture, we suspected that we could knock out SPC components directly without the need for a conditional KO or knockdown approach. We subsequently generated clonal homozygous KO strains of CP1, CP2, and MyoF and conducted endocytosis assays on the resulting mutants. Although we saw a reduction in endocytic rate in CP1 and CP2 KO strains, a much more significant decrease was observed in the Δ*MyoF* mutant and this defect was restored upon reintroduction of MyoF-Ty into the genome. However, because we had expected a complete loss of endocytosis in the Δ*MyoF* mutant, this led us to theorize that there may be additional myosin motors operating in either a redundant or compensatory capacity when MyoF is lost. The potential for myosin redundancy in endocytosis does have some precedence and has been observed in studies of *Arabidopsis* (37, 38). Our subsequent localization experiments revealed that, indeed, a total of four different myosin motors target to this organelle. Two of the myosins (MyoF and MyoC) target to the cytopharynx and two others (MyoB and MyoE) target to the POR (summarized in Fig. 7). As a result, it is possible that the cytopharyngeal MyoC may be responsible for facilitating the remaining endocytosis observed in the Δ*MyoF* mutant.

As we have noted previously, the plasma membrane at the POR gives the impression of a flowing river of membrane that emanates from the flagellar pocket and binds cargo at the parasite surface before descending into the cytostome itself, thus providing a mechanism for membrane homeostasis and receptor recycling (22, 23, 42, 43). This observation combined with the distinct localization of various proteins to this region implies that the POR may be an independently functioning subdomain of the SPC. In line with this thinking, the tightly restricted distribution of each myosin to subdomains of the SPC suggests that each subgroup potentially carries out independent and nonoverlapping functions. The myosin motors targeted to this POR region (MyoB and MyoE) may therefore function to propel bound surface cargo to the cytostome entrance, while those myosins on the tubular cytopharynx (MyoF and MyoC) could potentially serve to transport endocytosed vesicles to the posterior reservosomes for storage. Future combinatorial KO lines will no doubt be useful in determining the exact contribution of each myosin to these activities.

In summary, we have identified the first known molecular machinery involved in promoting the flow of endocytosed material into the cytostome-cytopharynx complex. To date, no clear descriptions of the mechanistic underpinnings that account for how this structure operates in any protozoan have been presented and this report provides the first evidence that the activity is driven, at least in part, through the action of myosin motors. Future studies will focus on discovering universal motifs or domains that drive various protein components to associate with the SPC and address what effect a loss of endocytosis would have on the ability of T. cruzi to propagate infection in both its arthropod and mammalian hosts.

## MATERIALS AND METHODS

### Parasite cultures.

Y strain epimastigotes were cultured in LDNT/LIT (liver digest neutralized tryptose/liver infusion tryptose) medium (57) that was supplemented with 15% heat (76°C)-inactivated (26) fetal bovine serum (FBS) (VWR, USDA certified). To obtain amastigotes, metacyclic trypomastigotes generated as previously described (26) were added to T25 flasks or coverslips containing confluent human foreskin fibroblasts (HFF). Infected monolayers were maintained in high-glucose Dulbecco’s modified Eagle’s medium (DMEM-HG) (HyClone) supplemented with l-glutamine and 1% heat (56°C)-inactivated Cosmic calf serum (CCS) (HyClone).

### Epimastigote growth assays.

Growth assays were performed as previously described (26). A total of 5 × 10^6^/ml epimastigotes of each strain were seeded into LIT media containing 15% heat-inactivated FBS. Counting was performed using a Coulter counter (Beckman Coulter).

### Generation of overexpression constructs and transfections.

For overexpression of mNeon-Ty tagged proteins, we utilized (i) the original pTREX vector (28) modified with the T. cruzi-optimized and “fixed” neomycin resistance cassette as previously described (26); (ii) the minimal pTREX vector that we had generated, pMiniTREX (see Fig. S4A in the supplemental material); or (iii) pTMiniTREX with the BBa_B1006 terminator (58) upstream of the insertion site (Fig. S4C). Final vectors were assembled using an NEB HiFi assembly kit. Parasites were transfected as described previously by Lander et al. (59) using a BTX ECM 830 system (Harvard Apparatus). However, only two electroporation pulses were used for transient transfections to reduce parasite loss for 48 h posttransfection imaging. A list of the DNA primers used during this work can be found in Table S1 in the supplemental material.

10.1128/mSphere.00313-20.7 TABLE S1 Primers used in this work. Download Table S1, PDF file, 0.1 MB. Copyright © 2020 Chasen et al. 2020 Chasen et al. This content is distributed under the terms of the Creative Commons Attribution 4.0 International license.

### Western blotting and immunofluorescence assays.

Western blotting and immunofluorescence assays (IFAs) were performed as previously described (26). Immunoblots were probed with anti-Ty mouse monoclonal antibody (MAb BB2) at 1:1,500, and loading controls were extracted from Stain-Free gel (Bio-Rad) total protein images. Anti-CP1 mouse polyclonal antibody (26) was used at a dilution of 1:500.

### Fluorescent microscopy.

Generation of mounted samples for fluorescence imaging was performed as previously described (26). Rhodamine-conjugated concanavalin A (Vector Laboratories) labeling of the preoral ridge was performed as described previously (26) using a concentration of 10 μg/ml. Images were obtained using the Zeiss Elyra S1 structured illumination microscope in the Center for Tropical and Emerging Diseases Biomedical Microscopy Core (BMC; Athens, GA).

### Sequence alignment and structure prediction.

T-Coffee (60) was used to generate sequence alignments, and alignment figures were produced using Jalview 2 software (61). Structure prediction of MyoF was generated using the I-TASSER server (62).

### Bioinformatic analysis of Trypanosoma cruzi genes.

Identification of potential SPC genes was performed using the EuPathDB (TriTrypDB) Orthology Phylogenetic Profile tool in the manner represented in Fig. 1. The recently assembled *T. cruzi* Y-strain genome (W. Wang et al., unpublished data) was used as the reference in all bioinformatic searches and experimental procedures.

### Flow cytometry-based endocytosis assays.

Epimastigotes growing in log phase were counted using a Beckman Coulter counter, and a total of 5 × 10^6^ cells were collected per sample and placed into 1.7-ml centrifuge tubes. Cells were pelleted at 1,000 × *g* at 4°C for 10 min. The supernatant was removed, and cells were resuspended in 500 μl of HBSS (Hanks balanced salt solution). Cytochalasin D (10 μM final concentration) was added to one sample 10 min prior to feeding as a negative control for endocytosis. Endocytosis was monitored by feeding parasites Alexa Fluor 647-conjugated BSA (Thermo Fisher) (final concentration of 10 μg/ml). Cells were subjected to brief vortex mixing and incubated at 28°C for 30 min in the dark. After feeding, each sample was transferred to 10 ml of HBSS as a wash. Cells were pelleted at 1,000 × *g* at 4°C for 10 min, the supernatant was removed, and the cells were resuspended in 1 ml of fresh HBSS and maintained on ice. Each flow cytometry sample was run using a Quanteon flow cytometer (Acea Bio) and analyzed using FlowJo.

### CRISPR/Cas9-mediated gene deletion and complementation.

Generation of deletion mutants was performed as described by Lander et al. (59) with modifications. The Cas9 and guide RNA (gRNA) regions were cloned from the single guide RNA (sgRNA)/Cas9/pTREX-n plasmid constructed by Lander et al. and inserted into a version of the pMiniTREX plasmid without a drug selection marker (Fig. S4B), for transient expression. Chosen protospacer regions were identified using the Eukaryotic Pathogen CRISPR guide RNA/DNA Design Tool server (http://grna.ctegd.uga.edu/) (63) and inserted into the vector using a NEB Q5 mutagenesis kit. Repair templates were generated by amplifying the blasticidin resistance gene that was cloned into PCR Blunt Topo II (Invitrogen) for this purpose. Primers for amplifying repair templates were generated containing the first 42 bp of the target gene’s coding region on the forward primer and the last 42 bp of the target gene’s coding region on the reverse primer. Amplification was performed in a 500 μl total PCR (50 μl each tube) using Q5 DNA polymerase (NEB). In order to generate complemented lines, repair templates for *MyoF-Ty* were generated in two steps. First, the MyoF gene was amplified and inserted into a pTMiniTrex plasmid containing the hygromycin resistance cassette. This construct was then used as a template for amplification of the repair fragment. This repair fragment was cotransfected with a pTMiniTrex-spCas9 plasmid (Fig. S4D) containing a guide RNA against the blasticidin cassette. Selection after transfection was performed as described for the knockouts with 250 μg/ml hygromycin instead of blasticidin. Transfections were performed as described above, using two electroporation pulses, and epimastigotes were maintained in 5 μg blasticidin (Gibco) starting 48 h after transfection. G418 (1,000 μg/ml) was also added 24 h after transfection and maintained for 7 days to stall epimastigote growth and maintain the presence of the transient Cas9-mNeon. After 7 days, epimastigotes were spun down and the medium was replaced with LIT containing only 5 μg blasticidin for outgrowth of deletion mutants. The transient Cas9-mNeon fluorescence was lost from the population during this outgrowth. Subcloning into 96-well plates was performed using a MoFlo Astrios EQ cell sorter in the Center for Tropical and Emerging Global Diseases Cytometry Shared Resource Laboratory (CSRL).

### Statistical analyses.

Statistical analyses for endocytosis assays were performed using the unpaired *t* test function of the Prism Software suite. Analyzed experiments were performed as three biological replicates. *P* values are denoted as follows: *, *P* < 0.05; **, *P* < 0.01; ***, *P* < 0.001.

## References

[1] Weekly Epidemiological Record. 2015 Chagas disease in Latin America: an epidemiological update based on 2010 estimates. Wkly Epidemiol Rec 90:33–43. 25671846

[2] Pérez-Molina JA, Molina I 2018 Chagas disease. Lancet 391:82–94. doi:10.1016/S0140-6736(17)31612-4. 28673423

[3] Groom ZC, Protopapas AD, Zochios V 2017 Tropical diseases of the myocardium: a review. Int J Gen Med 10:101–111. doi:10.2147/IJGM.S130828. 28435310 PMC5391162

[4] Camandaroba EL, Reis EA, Goncalves MS, Reis MG, Andrade SG 2003 Trypanosoma cruzi: susceptibility to chemotherapy with benznidazole of clones isolated from the highly resistant Colombian strain. Rev Soc Bras Med Trop 36:201–209. doi:10.1590/s0037-86822003000200002. 12806455

[5] Mejía-Jaramillo AM, Fernández GJ, Montilla M, Nicholls RS, Triana-Chávez O 2012 Trypanosoma cruzi strains resistant to benznidazole occurring in Colombia. Biomedica 32:196–205. (In Spanish.) 23242293 10.1590/S0120-41572012000300007

[6] Maguire JH 2015 Treatment of Chagas’ disease–time is running out. N Engl J Med 373:1369–1370. doi:10.1056/NEJMe1510170. 26323936

[7] Kansiime F, Adibaku S, Wamboga C, Idi F, Kato CD, Yamuah L, Vaillant M, Kioy D, Olliaro P, Matovu E 2018 A multicentre, randomised, non-inferiority clinical trial comparing a nifurtimox-eflornithine combination to standard eflornithine monotherapy for late stage Trypanosoma brucei gambiense human African trypanosomiasis in Uganda. Parasit Vectors 11:105. doi:10.1186/s13071-018-2634-x. 29471865 PMC5824494

[8] Abu Kwaik Y, Bumann D 2013 Microbial quest for food in vivo: ‘nutritional virulence’ as an emerging paradigm. Cell Microbiol 15:882–890. doi:10.1111/cmi.12138. 23490329

[9] Morgan GW, Hall BS, Denny PW, Carrington M, Field MC 2002 The kinetoplastida endocytic apparatus. Part I: a dynamic system for nutrition and evasion of host defences. Trends Parasitol 18:491–496. doi:10.1016/S1471-4922(02)02391-7. 12473365

[10] Morgan GW, Hall BS, Denny PW, Field MC, Carrington M 2002 The endocytic apparatus of the kinetoplastida. Part II: machinery and components of the system. Trends Parasitol 18:540–546. doi:10.1016/S1471-4922(02)02392-9. 12482539

[11] Field MC, Carrington M 2009 The trypanosome flagellar pocket. Nat Rev Microbiol 7:775–786. doi:10.1038/nrmicro2221. 19806154

[12] Flegontov P, Votypka J, Skalicky T, Logacheva MD, Penin AA, Tanifuji G, Onodera NT, Kondrashov AS, Volf P, Archibald JM, Lukes J 2013 Paratrypanosoma is a novel early-branching trypanosomatid. Curr Biol 23:1787–1793. doi:10.1016/j.cub.2013.07.045. 24012313

[13] Stevens JR 2014 Free-living bodonids and derived parasitic trypanosomatids: but what lies in between? Trends Parasitol 30:113–114. doi:10.1016/j.pt.2014.01.002. 24468209

[14] Flegontova O, Flegontov P, Malviya S, Poulain J, de Vargas C, Bowler C, Lukeš J, Horák A 2018 Neobodonids are dominant kinetoplastids in the global ocean. Environ Microbiol 20:878–889. doi:10.1111/1462-2920.14034. 29266706

[15] Skalický T, Dobáková E, Wheeler RJ, Tesařová M, Flegontov P, Jirsová D, Votýpka J, Yurchenko V, Ayala FJ, Lukeš J 2017 Extensive flagellar remodeling during the complex life cycle of Paratrypanosoma, an early-branching trypanosomatid. Proc Natl Acad Sci U S A 114:11757–11762. doi:10.1073/pnas.1712311114. 29078369 PMC5676924

[16] Goncalves CS, Avila AR, de Souza W, Motta MCM, Cavalcanti DP 2018 Revisiting the Trypanosoma cruzi metacyclogenesis: morphological and ultrastructural analyses during cell differentiation. Parasit Vectors 11:83. doi:10.1186/s13071-018-2664-4. 29409544 PMC5801705

[17] de Souza W, de Carvalho TM, Barrias ES 2010 Review on Trypanosoma cruzi: host cell interaction. Int J Cell Biol 2010:295394. doi:10.1155/2010/295394. 20811486 PMC2926652

[18] Barrias ES, de Carvalho TM, De Souza W 2013 Trypanosoma cruzi: entry into mammalian host cells and parasitophorous vacuole formation. Front Immunol 4:186. doi:10.3389/fimmu.2013.00186. 23914186 PMC3730053

[19] Alcantara CL, Vidal JC, de Souza W, Cunha E 2017 The cytostome-cytopharynx complex of Trypanosoma cruzi epimastigotes disassembles during cell division. J Cell Sci 130:164–176. doi:10.1242/jcs.187419. 27363990

[20] Vidal JC, Alcantara CL, de Souza W, Cunha E 2016 Loss of the cytostome-cytopharynx and endocytic ability are late events in Trypanosoma cruzi metacyclogenesis. J Struct Biol 196:319–328. doi:10.1016/j.jsb.2016.07.018. 27480509

[21] Alcantara CL, Vidal JC, de Souza W, Cunha-e-Silva NL 2014 The three-dimensional structure of the cytostome-cytopharynx complex of Trypanosoma cruzi epimastigotes. J Cell Sci 127:2227–2237. doi:10.1242/jcs.135491. 24610945

[22] Martinez-Palomo A, DeSouza W, Gonzalez-Robles A 1976 Topographical differences in the distribution of surface coat components and intramembrane particles. A cytochemical and freeze-fracture study in culture forms of Trypanosoma cruzi. J Cell Biol 69:507–513. doi:10.1083/jcb.69.2.507. 770483 PMC2109674

[23] Vatarunakamura C, Ueda-Nakamura T, de Souza W 2005 Visualization of the cytostome in Trypanosoma cruzi by high resolution field emission scanning electron microscopy using secondary and backscattered electron imaging. FEMS Microbiol Lett 242:227–230. doi:10.1016/j.femsle.2004.11.008. 15621442

[24] Souto-Padrón T, de Souza W 1983 Freeze-fracture localization of filipin-cholesterol complexes in the plasma membrane of Trypanosoma cruzi. J Parasitol 69:129–137. doi:10.2307/3281287. 6402578

[25] Steverding D, Stierhof YD, Chaudhri M, Ligtenberg M, Schell D, Beck-Sickinger AG, Overath P 1994 ESAG 6 and 7 products of Trypanosoma brucei form a transferrin binding protein complex. Eur J Cell Biol 64:78–87. 7957316

[26] Chasen NM, Coppens I, Etheridge RD 2019 Identification and localization of the first known proteins of the Trypanosoma cruzi cytostome cytopharynx endocytic complex. Front Cell Infect Microbiol 9:445. doi:10.3389/fcimb.2019.00445. 32010635 PMC6978632

[27] de Souza DAS, Pavoni DP, Krieger MA, Ludwig A 2018 Evolutionary analyses of myosin genes in trypanosomatids show a history of expansion, secondary losses and neofunctionalization. Sci Rep 8:1376. doi:10.1038/s41598-017-18865-y. 29358582 PMC5778035

[28] Martínez-Calvillo S, López I, Hernández R 1997 pRIBOTEX expression vector: a pTEX derivative for a rapid selection of Trypanosoma cruzi transfectants. Gene 199:71–76. doi:10.1016/S0378-1119(97)00348-X. 9358041

[29] Godsel LM, Tibbetts RS, Olson CL, Chaudoir BM, Engman DM 1995 Utility of recombinant flagellar calcium-binding protein for serodiagnosis of Trypanosoma cruzi infection. J Clin Microbiol 33:2082–2085. doi:10.1128/JCM.33.8.2082-2085.1995. 7559952 PMC228339

[30] Andre J, Kerry L, Qi X, Hawkins E, Drizyte K, Ginger ML, McKean PG 2014 An alternative model for the role of RP2 protein in flagellum assembly in the African trypanosome. J Biol Chem 289:464–475. doi:10.1074/jbc.M113.509521. 24257747 PMC3879569

[31] Chen CK, Chan NL, Wang AH 2011 The many blades of the beta-propeller proteins: conserved but versatile. Trends Biochem Sci 36:553–561. doi:10.1016/j.tibs.2011.07.004. 21924917

[32] Pasha SN, Meenakshi I, Sowdhamini R 2016 Revisiting myosin families through large-scale sequence searches leads to the discovery of new myosins. Evol Bioinform Online 12:201–211. doi:10.4137/EBO.S39880. 27597808 PMC5006635

[33] Li X-d, Rhodes TE, Ikebe R, Kambara T, White HD, Ikebe M 1998 Effects of mutations in the γ-phosphate binding site of myosin on its motor function. J Biol Chem 273:27404–27411. doi:10.1074/jbc.273.42.27404. 9765269

[34] Bogitsh BJ, Ribeiro-Rodrigues R, Carter CE 1995 In vitro effects of mannan and cytochalasin B on the uptake of horseradish peroxidase and [14C]sucrose by Trypanosoma cruzi epimastigotes. J Parasitol 81:144–148. doi:10.2307/3283912. 7707187

[35] Correa JR, Atella GC, Batista MM, Soares MJ 2008 Transferrin uptake in Trypanosoma cruzi is impaired by interference on cytostome-associated cytoskeleton elements and stability of membrane cholesterol, but not by obstruction of clathrin-dependent endocytosis. Exp Parasitol 119:58–66. doi:10.1016/j.exppara.2007.12.010. 18234197

[36] Lander N, Chiurillo MA, Docampo R 2019 Genome editing by CRISPR/Cas9 in Trypanosoma cruzi. Methods Mol Biol 1955:61–76. doi:10.1007/978-1-4939-9148-8_5. 30868519

[37] Prokhnevsky AI, Peremyslov VV, Dolja VV 2008 Overlapping functions of the four class XI myosins in Arabidopsis growth, root hair elongation, and organelle motility. Proc Natl Acad Sci U S A 105:19744–19749. doi:10.1073/pnas.0810730105. 19060218 PMC2596744

[38] Ojangu EL, Tanner K, Pata P, Jarve K, Holweg CL, Truve E, Paves H 2012 Myosins XI-K, XI-1, and XI-2 are required for development of pavement cells, trichomes, and stigmatic papillae in Arabidopsis. BMC Plant Biol 12:81. doi:10.1186/1471-2229-12-81. 22672737 PMC3424107

[39] Au JS, Puri C, Ihrke G, Kendrick-Jones J, Buss F 2007 Myosin VI is required for sorting of AP-1B-dependent cargo to the basolateral domain in polarized MDCK cells. J Cell Biol 177:103–114. doi:10.1083/jcb.200608126. 17403927 PMC2064115

[40] Lister IM, Tolliday NJ, Li R 2006 Characterization of the minimum domain required for targeting budding yeast myosin II to the site of cell division. BMC Biol 4:19. doi:10.1186/1741-7007-4-19. 16800887 PMC1559645

[41] He F, Wollscheid HP, Nowicka U, Biancospino M, Valentini E, Ehlinger A, Acconcia F, Magistrati E, Polo S, Walters KJ 2016 Myosin VI contains a compact structural motif that binds to ubiquitin chains. Cell Rep 14:2683–2694. doi:10.1016/j.celrep.2016.01.079. 26971995 PMC4805485

[42] de Souza W, Sant'Anna C, Cunha-e-Silva NL 2009 Electron microscopy and cytochemistry analysis of the endocytic pathway of pathogenic protozoa. Prog Histochem Cytochem 44:67–124. doi:10.1016/j.proghi.2009.01.001. 19410686

[43] De Souza W, Martínez-Palomo A, González-Robles A 1978 The cell surface of Trypanosoma cruzi: cytochemistry and freeze-fracture. J Cell Sci 33:285–299. 363731 10.1242/jcs.33.1.285

[44] Arden SD, Tumbarello DA, Butt T, Kendrick-Jones J, Buss F 2016 Loss of cargo binding in the human myosin VI deafness mutant (R1166X) leads to increased actin filament binding. Biochem J 473:3307–3319. doi:10.1042/BCJ20160571. 27474411 PMC5074368

[45] Hamedi A, Botelho L, Britto C, Fragoso SP, Umaki AC, Goldenberg S, Bottu G, Salmon D 2015 In vitro metacyclogenesis of Trypanosoma cruzi induced by starvation correlates with a transient adenylyl cyclase stimulation as well as with a constitutive upregulation of adenylyl cyclase expression. Mol Biochem Parasitol 200:9–18. doi:10.1016/j.molbiopara.2015.04.002. 25912925

[46] Vidal JC, Alcantara CDL, DE Souza W, Cunha-E-Silva NL 2017 Lysosome-like compartments of Trypanosoma cruzi trypomastigotes may originate directly from epimastigote reservosomes. Parasitology 144:841–850. doi:10.1017/S0031182016002602. 28077187

[47] Prats C, Graham TE, Shearer J 2018 The dynamic life of the glycogen granule. J Biol Chem 293:7089–7098. doi:10.1074/jbc.R117.802843. 29483195 PMC5949993

[48] Sugi T, Tu V, Ma Y, Tomita T, Weiss LM 2017 Toxoplasma gondii requires glycogen phosphorylase for balancing amylopectin storage and for efficient production of brain cysts. mBio 8:e01289-17. doi:10.1128/mBio.01289-17. 28851850 PMC5574715

[49] Uboldi AD, McCoy JM, Blume M, Gerlic M, Ferguson DJ, Dagley LF, Beahan CT, Stapleton DI, Gooley PR, Bacic A, Masters SL, Webb AI, McConville MJ, Tonkin CJ 2015 Regulation of starch stores by a Ca(2+)-dependent protein kinase is essential for viable cyst development in Toxoplasma gondii. Cell Host Microbe 18:670–681. doi:10.1016/j.chom.2015.11.004. 26651943

[50] Soares MJ, De Souza W 1988 Cytoplasmic organelles of trypanosomatids: a cytochemical and stereological study. J Submicrosc Cytol Pathol 20:349–361. 3135113

[51] Soares MJ, de Souza W 1991 Endocytosis of gold-labeled proteins and LDL by Trypanosoma cruzi. Parasitol Res 77:461–468. doi:10.1007/BF00928410. 1656428

[52] Soares MJ, Souto-Padrón T, De Souza W 1992 Identification of a large pre-lysosomal compartment in the pathogenic protozoon Trypanosoma cruzi. J Cell Sci 102:157–167. 1500438 10.1242/jcs.102.1.157

[53] Figueiredo RC, Steindel M, Soares MJ 1994 The reservosomes of epimastigote forms of Trypanosoma cruzi: occurrence during in vitro cultivation. Parasitol Res 80:517–522. doi:10.1007/BF00932700. 7809003

[54] Vanrell MC, Losinno AD, Cueto JA, Balcazar D, Fraccaroli LV, Carrillo C, Romano PS 2017 The regulation of autophagy differentially affects Trypanosoma cruzi metacyclogenesis. PLoS Negl Trop Dis 11:e0006049. doi:10.1371/journal.pntd.0006049. 29091711 PMC5683653

[55] Barison MJ, Rapado LN, Merino EF, Furusho Pral EM, Mantilla BS, Marchese L, Nowicki C, Silber AM, Cassera MB 2017 Metabolomic profiling reveals a finely tuned, starvation-induced metabolic switch in Trypanosoma cruzi epimastigotes. J Biol Chem 292:8964–8977. doi:10.1074/jbc.M117.778522. 28356355 PMC5448128

[56] Ferreira LRP, Dossin FDM, Ramos TC, Freymüller E, Schenkman S 2008 Active transcription and ultrastructural changes during Trypanosoma cruzi metacyclogenesis. An Acad Bras Cienc 80:157–166. doi:10.1590/s0001-37652008000100011. 18345384

[57] Kirchhoff LV, Hieny S, Shiver GM, Snary D, Sher A 1984 Cryptic epitope explains the failure of a monoclonal antibody to bind to certain isolates of Trypanosoma cruzi. J Immunol 133:2731–2735. 6207242

[58] Huang H 2007 Design and characterization of artificial transcriptional terminators. Massachusetts Institute of Technology, Cambridge, MA https://dspace.mit.edu/handle/1721.1/45981.

[59] Lander N, Li ZH, Niyogi S, Docampo R 2015 CRISPR/Cas9-induced disruption of paraflagellar rod protein 1 and 2 genes in Trypanosoma cruzi reveals their role in flagellar attachment. mBio 6:e01012. doi:10.1128/mBio.01012-15. 26199333 PMC4513075

[60] Madeira F, Park YM, Lee J, Buso N, Gur T, Madhusoodanan N, Basutkar P, Tivey ARN, Potter SC, Finn RD, Lopez R 2019 The EMBL-EBI search and sequence analysis tools APIs in 2019. Nucleic Acids Res 47:W636–W641. doi:10.1093/nar/gkz268. 30976793 PMC6602479

[61] Waterhouse AM, Procter JB, Martin DM, Clamp M, Barton GJ 2009 Jalview Version 2–a multiple sequence alignment editor and analysis workbench. Bioinformatics 25:1189–1191. doi:10.1093/bioinformatics/btp033. 19151095 PMC2672624

[62] Yang J, Zhang Y 2015 I-TASSER server: new development for protein structure and function predictions. Nucleic Acids Res 43:W174–W181. doi:10.1093/nar/gkv342. 25883148 PMC4489253

[63] Peng D, Tarleton R 2015 EuPaGDT: a Web tool tailored to design CRISPR guide RNAs for eukaryotic pathogens. Microb Genom 1:e000033. doi:10.1099/mgen.0.000033. 28348817 PMC5320623

