# Supplementary figures and images for: The Functional Characterization of TcMyoF Implicates a Family of Cytostome-Cytopharynx Targeted Myosins as Integral to the Endocytic Machinery of Trypanosoma cruzi
Source: mSphere. 2020 Jun 17;5(3):e00313-20. doi: 10.1128/mSphere.00313-20 (PMC7300353; doi:10.1128/mSphere.00313-20)

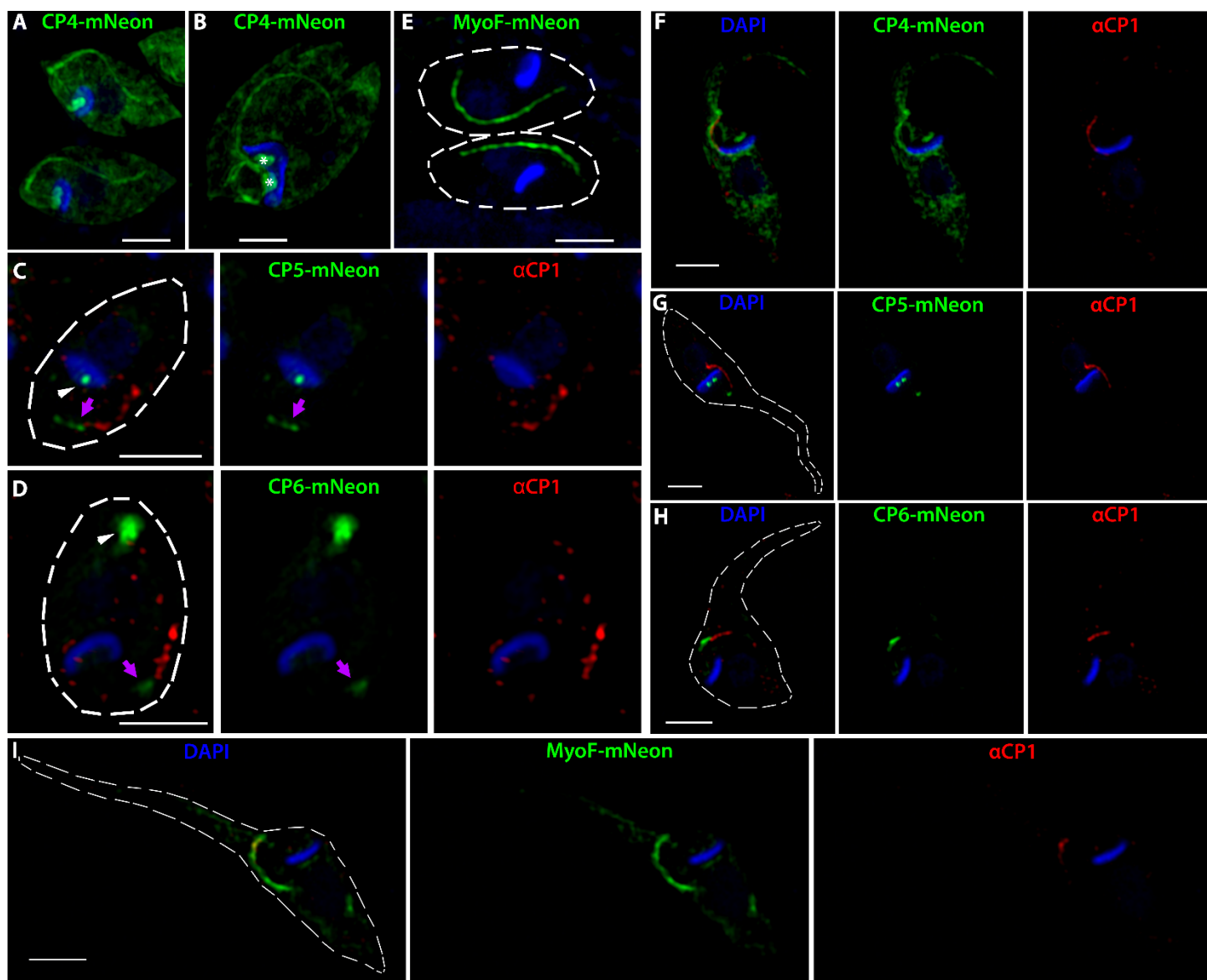

Supplement: FIG S1 [file mSphere.00313-20-sf001.pdf]

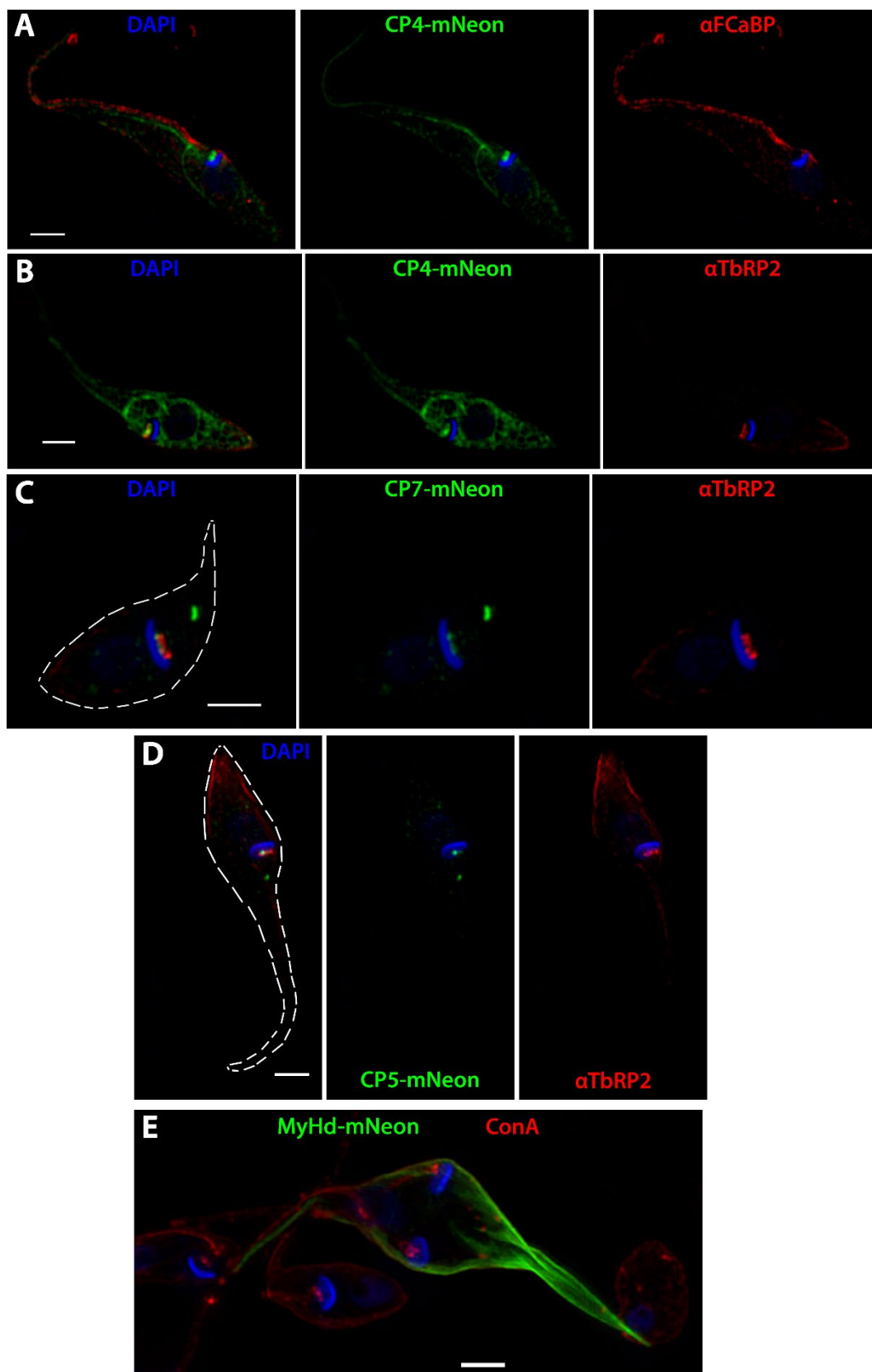

Supplement: FIG S2 [file mSphere.00313-20-sf002.pdf]

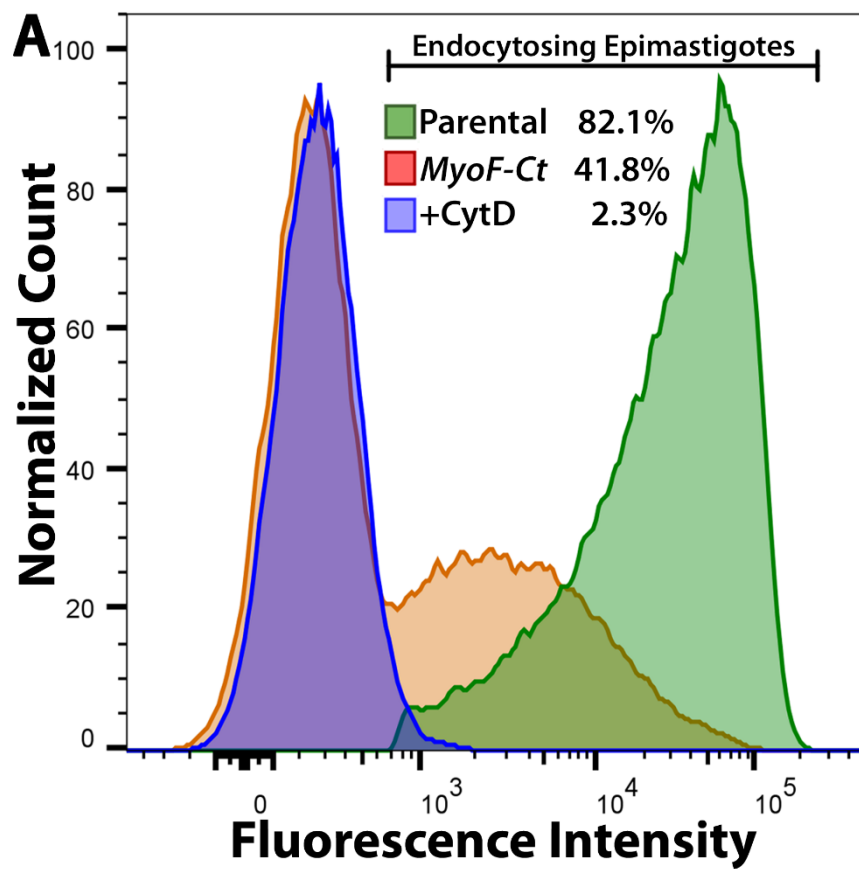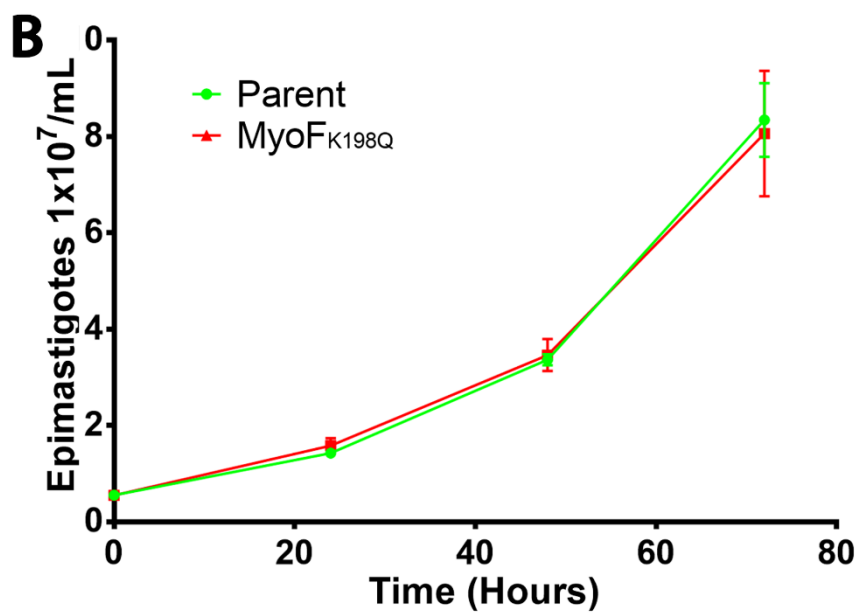

Supplement: FIG S3 [file mSphere.00313-20-sf003.pdf]

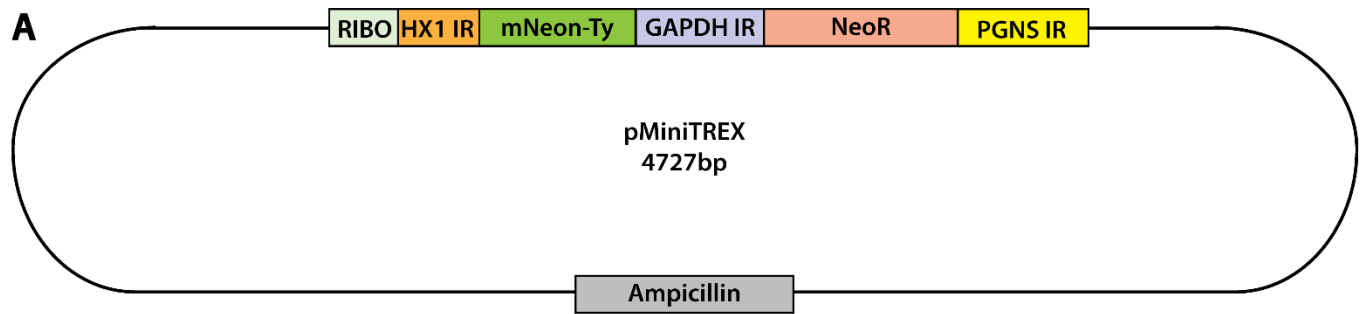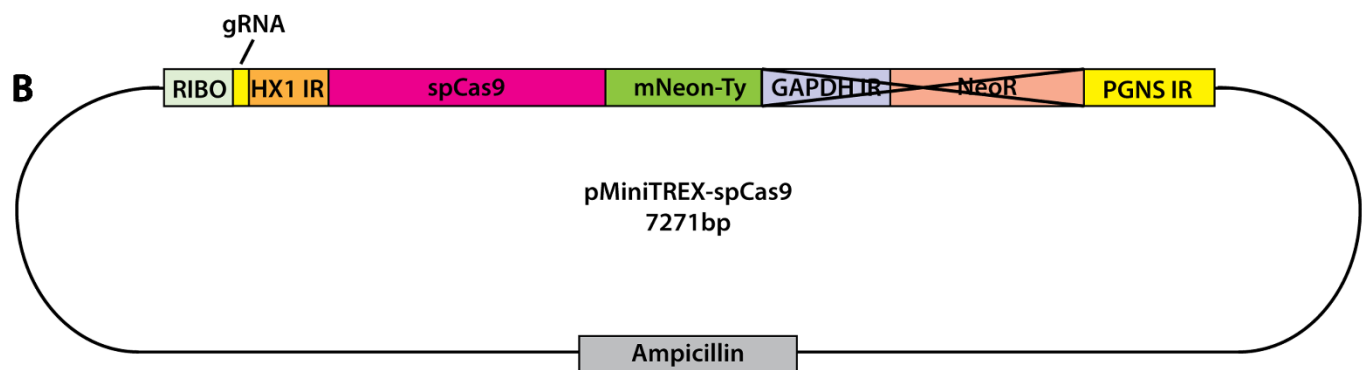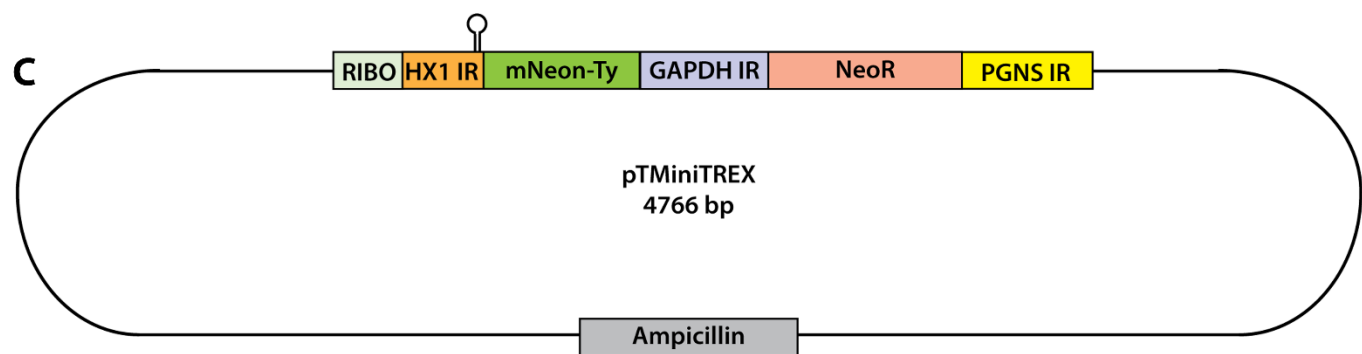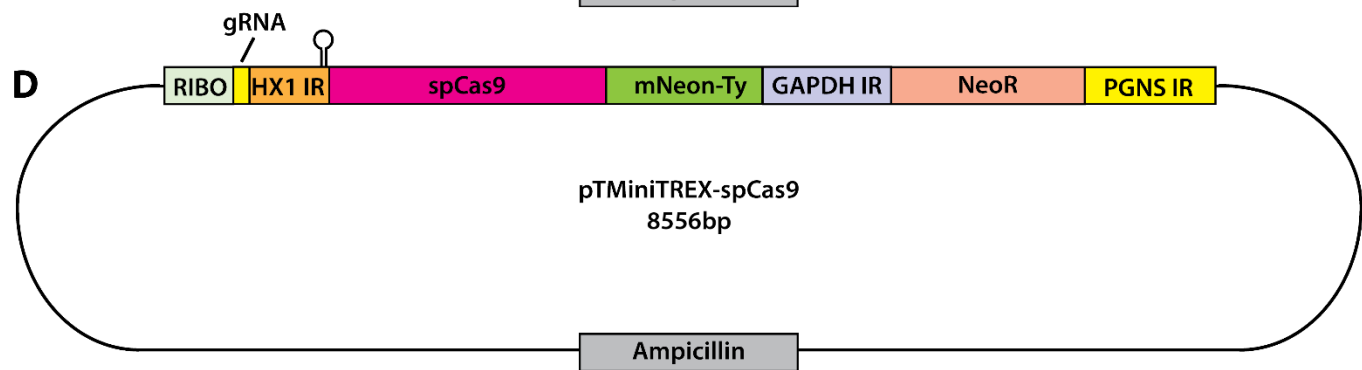

Supplement: FIG S4 [file mSphere.00313-20-sf004.pdf]

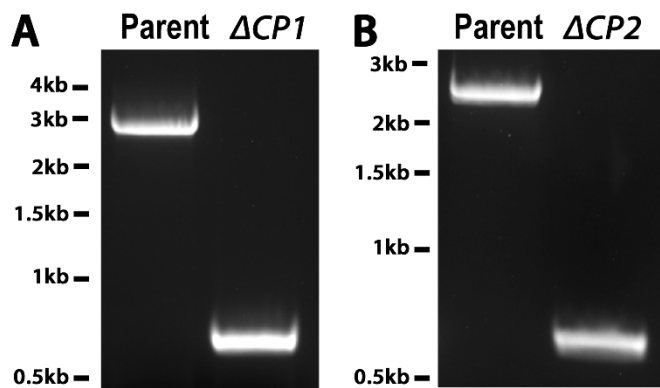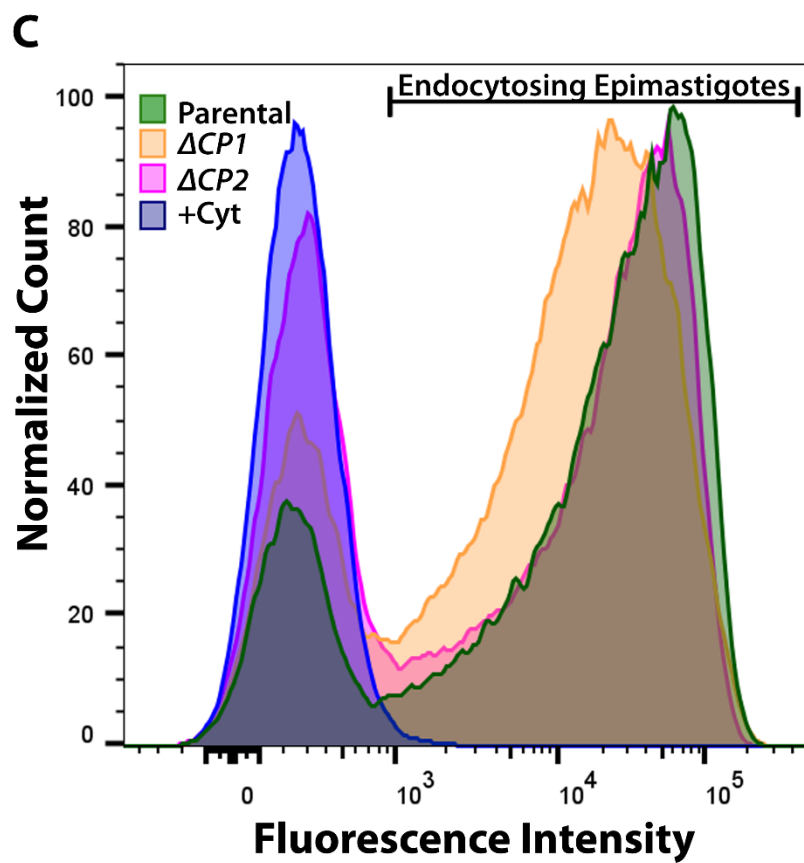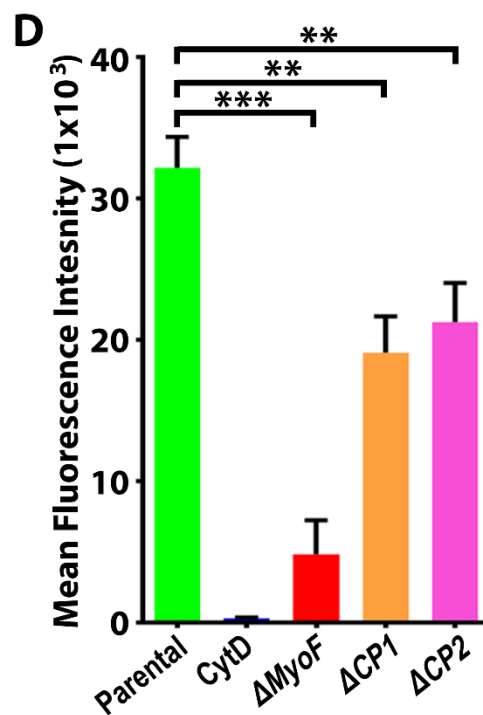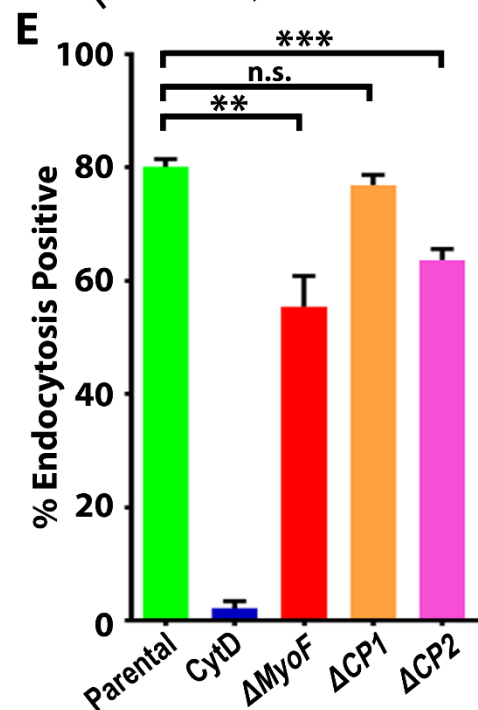

Supplement: FIG S5 [file mSphere.00313-20-sf005.pdf]

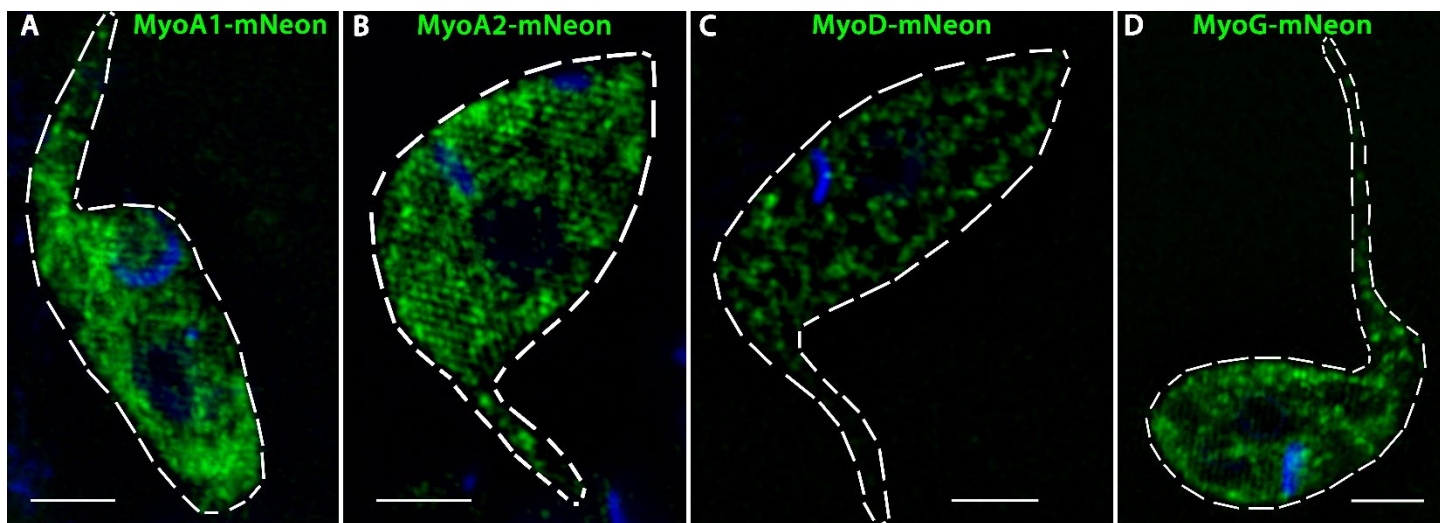

Supplement: FIG S6 [file mSphere.00313-20-sf006.pdf]
