# Supplementary material for: The Functional Characterization of TcMyoF Implicates a Family of Cytostome-Cytopharynx Targeted Myosins as Integral to the Endocytic Machinery of Trypanosoma cruzi
Source: mSphere. 2020 Jun 17;5(3):e00313-20. doi: 10.1128/mSphere.00313-20 (PMC7300353; doi:10.1128/mSphere.00313-20)
Supplement: TABLE S1 [file mSphere.00313-20-st001.pdf]

Table of Primers Used in This Work

**Protospacer Mutagenesis and Repair**			
Universal Protospacer Q5 Mutagenesis Reverse	MT28	GGATCCACTAGAACTCTTGCACAGACTTGGC	antisense
CP1 Protospacer Mutagenesis Forward	MT18	GTACCCGTTTCATCCTGTCAgttttagagctagaaatagcaagttaaaataaggctag	sense
CP2 Protospacer Mutagenesis Forward	MT30	GTGCTCGAGAGGAACAGCAGgttttagagctagaaatagcaagttaaaataaggctag	sense
MyoF Protospacer Mutagenesis Forward	MT13	ATTCAGAGTCGTGTGTTCTGgttttagagctagaaatagcaagttaaaataaggc	sense
CP1 Blasticidin Repair Template Forward	MT21	ATGAATGGGGGCGATGACGACGCCCACAACGCCTCAGTGATCgccaagcctttgtctcaa	sense
CP1 Blasticidin Repair Template Reverse	MT22	AGCAACGGTGTCGTCCTCTTCTAATTCCATCTCCCACTTGCTttagccctcccacacata	antisense
CP2 Blasticidin Repair Template Forward	MT31	ATGTCGCATGAAAGCAGTCCTTTGCAGGTGGGCTCCGCTTTAgccaagcctttgtctcaa	sense
CP2 Blasticidin Repair Template Reverse	MT32	TAAGGATTGTAACGATAAGAGCTCCCGTTCGTCCTCCTCAttagccctcccacacataac	antisense
MyoF Blasticidin Repair Template Forward	MT17	ATGGCGGAGGCTTCTGTAACATCCCACGATGGCTATTGTGTGgccaagcctttgtctcaa	sense
MyoF Blasticidin Repair Template Reverse	MT16	ACGACTAAAGCTAGGAAAATATGGCTCGGGTGAATTCCCGCAttagccctcccacacata	antisense
CP1 Deletion Screen Forward	MT77	GTGACTTGTGAGTTTACAAGGAGGGAGAGAGAG	sense
CP1 Deletion Screen Reverse	MT78	GGTTCACATTTCACACAGGTGTATACCGTGC	antisense
CP2 Deletion Screen Forward	MT75	GCCACTTTAAAGAAGCGCAAGGTTGAGG	sense
CP2 Deletion Screen Reverse	MT76	CAACAGTCATCAAATACAGGCATACACGCAAACG	antisense

MyoF Deletion Screen Forward	MT79	GAGGAAGTGCCAAAAGTTTGGTGACAGG	sense
MyoF Deletion Screen Reverse	MT80	CGGTATAAATGTGTTTCCTATACGATAAATACGATAGCGATACTCAAC	antisense
MyoF-Ty Repair Template Forward	CS97	ATGGCGGAGGTTTCTGTAAAATCCCACGATGGCTATTGTGTG	sense
MyoF-Ty Repair Template Reverse	CS98	TACTAAACAAAAACCTAACGACTAAAGCTAGTAAAATACGCTATTCCTTTGCCCTCGGAC	antisense
**Assembly of pMiniTREX Variants**			
Ampicillin F for miniTrex	PZ87	tctcaagaagatcctttgatcttttctacgTTACCAATGCTTAATCAGTGAGGC	sense
Ampicillin R for miniTrex	PZ29	CACCAAATAAGCCCCGCAAGGAAACTGGTGtatgtatccgctcatgagacaataac	antisense
picoZ Origin Region Forward	PZ28	GTCGCCGCGGGTACCTCGTGTGCACGGCTGgtttttccataggctccgcc	sense
picoz Origin Region Reverse	PZ 67	cgtagaaaagatcaaaggatcttcttgagatcc	antisense
Ribosomal Promoter and HX1 Forward	PZ23	CACCAGTTTCCTTGCGG	sense
Ribosomal Promoter and HX1 Reverse	CS59	CATTCTAGACAAGACAACTTATAGAGC	antisense
mNeonTy Forward	PZ24	GCTCTATAAGTTGTCTTGTCTAGAATGGTGTCCAAAGGAGAAGAAGACA	sense
mNeonTy Reverse	PZ83	CAAAAGAAAGACCTGAAGTCATCGCCACGCTTAATCAAGGGGATCTTGATTAGTATGCAC	antisense
minimal GAPDH Forward	PZ34	GCGTGGCGATGACTTCAGGTCTTTCTTTTGC	sense
minimal GAPDH Reverse	PZ35	AATGCACAAACACTAAACTACACCTTAAACACCATCAGTGAAG	antisense
Neomycin Resistance Cassette Forward	PZ82	GTTTAAGGTGTAGTTTAGTGTTTGTGCATTATGATTGAACAGGACGGACTC	sense
Neomycin Resistance Cassette Reverse	PZ27	ACAAATATAATGAGAGTAGAGGTATCCGTGTCAAAAGAATTCGTCCAGGAGAC	antisense
F2Postaglandin Synthase 3'UTR Forward	PZ3	CACGGATACCTCTACTCTCATTATATTTGTCTTTTTTTATCAAAATAAGCACGGATTAGC	sense
F2Postaglandin Synthase 3'UTR Forward	PZ4	CAGCCGTGCACACGAGGTACCC	antisense

miniTrex Forward for guideRNA Insertion	SE8	CCCAACGAGTTTCTTCAAAATATGCAGC	sense
miniTrex Reverse for guideRNA Insertion	SE7	GGATCCACTAGAACTCTTGCACAGAC	antisense
guideRNA Forward	MT24	GTCTGTGCAAGAGTTCTAGTGGATCCgttttagagctagaaatagcaagttaaaataagg	sense
guideRNA Reverse	MT25	CTGCATATTTTGAAGAAACTCGTTGGGggatccaaaaaagcaccgactc	antisense
HX1 Reverse for spCas9 Insertion	CS59	CATTCTAGACAAGACAACTTATAGAGC	antisense
mNeon Forward for spCas9 Insertion	NC93	GTGTCCAAAGGAGAAGAAGAC	sense
spCas9 Forward for miniTrex Insertion	PZ48	TCTATAAGTTGTCTTGTCTAGAATGgacaagaagtacagcatcggcctggacatcg	sense
spCas9 Reverse for miniTrex Insertion	PZ47	TCTTCTTCTCCTTTGGACACagatccgctgctgccgttgctcc	antisense
Forward for Bba_B1006 Terminator Insertion	MT92	GGGTTTTTTTTTGTCTAGAATGGTGTCCAAAGGAGAAGAAGAC	sense
Reverse for Bba_B1006 Terminator Insertion	MT91	GGGGTTTTTTTTTAGACAACTTATAGAGCAAAAAAAAAAGAAAAGACATCATAAAAGAAG	antisense
Bba_B1006 Terminator Duplex Sense Strand	PZ94	AAGTTGTCTAAAAAAAAACCCCGCCCCTGACAGGGCGGGGTTTTTTTTTGTCTAGAATGG	sense
Bba_B1006 Terminator Duplex Anti-Sense Strand	PZ95	CCATTCTAGACAAAAAAAAACCCCGCCCTGTCAGGGGCGGGGTTTTTTTTTAGACAACTT	antisense
**Myosin C-termini Constructs**			
GAPDH Universal Forward for Myo C-t tests	PZ24	GCGTGGCGATGACTTCAGGTCTTTCTTTTGC	sense
mNeon-Ty Universal Reverse for Myo C-t tests	N21	GAAGTGCATACTAATCAAGATCCCCTTGAT	antisense
MyoF C-t Forward for pMiniTrex	CS76	GCATACTAATCAAGATCCCCTTGATCAGGATGCTTTACGAAGGGAAAAGG	sense

MyoF C-t Reverse for pMiniTrex	CS77	GAAAGACCTGAAGTCATCGCCACGCTTAACGACTAAAGCTAGTAAAATACGGCTCG	antisense
MyoF C-tFirst Part Reverse for pMiniTrex	CS78	CAAAAGAAAGACCTGAAGTCATCGCCACGCTTACCCGAGCAACGAAGGTGACC	antisense
MyoF C-t Second Part Forward for pMiniTrex	CS79	GCATACTAATCAAGATCCCCTTGATCCTGCAGGTTTTATCAAGGCG	sense
MyoC Yqik Forward for pMiniTrex	CS74	GCATACTAATCAAGATCCCCTTGATTTGAAAAGCGTTGGATTTGACTCC	sense
MyoC Yqik Reverse for pMiniTrex	CS83	CAAAAGAAAGACCTGAAGTCATCGCCACGCTTAACGTCTCTGCGCCTCTCG	antisense
MyoC C-t Forward for pMiniTrex	CS82	GCATACTAATCAAGATCCCCTTGATGAGGCAGAAAATGCTCGTCTAGAGG	sense
MyoC C-t Reverse for pMiniTrex	CS75	GAAAGACCTGAAGTCATCGCCACGCTTAAAAACCAAAGTCCATCGTACTTTGC	antisense
MyoB C-t Forward for pMiniTrex	CS84	GCATACTAATCAAGATCCCCTTGATCATTGTGAAGCAATTCGGTTGGC	sense
MyoB C-t Reverse for pMiniTrex	CS85	CAAAAGAAAGACCTGAAGTCATCGCCACGCTTACGCCCAGCGTTTTGAGCG	antisense
MyoE C-t Forward for pMiniTrex	CS86	GCATACTAATCAAGATCCCCTTGATCGGTATTTGCTTTGTACATCACTGATTATTCAG	sense
MyoE C-t Reverse for pMiniTrex	CS87	CAAAAGAAAGACCTGAAGTCATCGCCACGCTTATTGCTCTGATCCAAACACGCTATCC	antisense
